# Training Medical Students as Peer-Facilitators to Identify Medical Student Mistreatment in the Clerkship Year

**DOI:** 10.15766/mep_2374-8265.11185

**Published:** 2021-09-27

**Authors:** Martine N. Randolph, Emily Cokorinos Erb, Priya S. Garg, Rachel Thompson, Molly Cohen-Osher

**Affiliations:** 1 Family Medicine Resident, MedStar Health Franklin Square; 2 Internal Medicine Resident, Beth Israel Deaconess Medical Center; 3 Assistant Professor, Department of Pediatrics, Boston University School of Medicine; Associate Dean of Medical Education, Boston University School of Medicine; 4 Assistant Professor, Department of Pediatrics, Boston University School of Medicine; 5 Assistant Professor, Department of Family Medicine and Medical Sciences and Education, Boston University School of Medicine; Assistant Dean of Curriculum and Instructional Design, Boston University School of Medicine

**Keywords:** Quality Improvement/Patient Safety, Well-Being/Mental Health, Program Evaluation

## Abstract

**Introduction:**

Data from the Association of American Medical Colleges' Medical School Graduation Questionnaire show persistent trends of medical student mistreatment nationwide. To reduce the barriers and increase actionable reporting of mistreatment, we integrated peer-facilitated learning environment sessions led by a group of trained third- and fourth-year medical students in all core clinical clerkships.

**Methods:**

During the 2018–2019 academic year, third-year medical students were recruited, oriented, and trained to act as facilitators of sessions on mistreatment. The sessions occurred once every clerkship block, using a standardized session introduction and guide. After a 6-month pilot, new medical students were recruited and worked as scribe/facilitator pairs, receiving an additional 1.5-hour training midyear, which was evaluated with a postworkshop survey.

**Results:**

Thirty-eight students implemented 43 peer-facilitated sessions and completed deidentified minutes of each session, which were shared with clerkship directors and the Medical Education Office for review. Survey data from midyear facilitator training indicated that facilitators highly agreed peer-led sessions were an important avenue for students to process experiences of mistreatment (3.9 out of 4), understood barriers to reporting (3.8 out of 4) and definitions of mistreatment (3.6 out of 4), and felt confident to facilitate these sessions (3.6 out of 4).

**Discussion:**

Peer-facilitated sessions offer a method to learn more about student experiences with mistreatment in real time and create a new avenue for communication between faculty and students. Assembling a stable core team of third- and fourth-year students trained in facilitation skills ensures the sustainability and relevance of the program.

## Educational Objectives

By the end of this session, student facilitators will be able to:

1. Define student mistreatment.
2. Describe the importance of peer-facilitated debriefing sessions.
3. Facilitate solutions-oriented discussions with peers and troubleshoot common scenarios of mistreatment or unfair treatment.
4. Provide support to clerkship students in a group setting.
5. Document anonymous and actionable feedback to clerkship directors about strengths and opportunities to improve the learning environment in clinical clerkships.

## Introduction

Medical student mistreatment in clinical clerkships persists despite institution-wide efforts to better protect students. According to a recent study, 35% of all medical students reported experiencing at least one type of mistreatment.^1^ Medical schools recognize the imperative to eradicate mistreatment given its deleterious effects on student mental health and on satisfaction with career choice.^2–6^ At the Boston University School of Medicine, multiple formal mechanisms are in place for anonymous and confidential reporting of mistreatment. These include anonymous student evaluations in the clerkship year with a specific open-ended question related to mistreatment, annual learning environment surveys featuring a series of mistreatment-related questions, and an anonymous mistreatment reporting site reviewed monthly by the Appropriate Treatment in Medicine (ATM) committee. We also annually present to all students about these reporting channels and the institutional policies on mistreatment. This has led to increased knowledge of ways to report mistreatment and has identified some incidences of mistreatment but unfortunately has not resulted in an increased number of student reports in congruence with the number reported in the graduation questionnaire administered by the Association of American Medical Colleges at the end of medical school.^7^ This disparity between the number of mistreatment instances identified by students during medical school, particularly in the clerkship year, and the number of instances identified long after they have happened has hindered our institution's ability to reform the learning environment in proximity to the mistreatment events students are experiencing.

Most medical schools have approached addressing mistreatment by increasing students' knowledge of what defines mistreatment and their recognition of potential mistreatment scenarios before they enter the clinical environment. Published interventions have included creating case and video vignettes, multimedia modules, and interactive workshops with mistreatment scenarios.^8–10^ Although it is important that students are given hypothetical and simulated mistreatment scenarios before entering the clinical environment, we believe that the challenge for clerkship students is identifying mistreatment while they are facing it and feeling safe and empowered to report it. Despite our efforts to embed a mistreatment workshop during our third-year clerkship orientation, as well as clerkship directors' continued encouragement to report any issues or concerns about mistreatment to them, write about mistreatment in the end-of-clerkship evaluation, or report mistreatment to the ATM committee, our clerkship students described the need to debrief and discuss mistreatment with others in real time during the clerkship year to reduce hesitancy to report and increase their perceived safety through anonymous group reporting.

Therefore, our medical school turned to near-peer teaching and group facilitation as an approach to lower barriers to reporting mistreatment.^8^ Peer-led facilitation has been shown to be preferred by students and to increase participation when not in the presence of faculty.^11,12^ We hypothesized that by creating peer-led learning environment sessions, students would be able to better define and identify mistreatment behaviors and create therapeutic alliances among their peers to discuss and debrief scenarios they faced. In addition, peer-led facilitation would reduce barriers to reporting, lead to earlier identification of mistreatment, and add specificity to scenarios faced by students in the clerkship year. To facilitate successful peer-led sessions, we identified student peer-facilitators and created a training workshop focused on facilitating discussion about the clerkship learning environment and mistreatment.

## Methods

### Student Facilitator Recruitment and Assignments

Third-year student facilitators were recruited through an online application (Appendix A) several weeks into the start of their clerkship year. Before assigning third-year students to groups, we asked them about their future plans for residency and did not assign them to their specialty of interest to avoid conflicts of interest. We divided student facilitators into three groups. Each group of students covered two to three clerkships for continuity with clerkship directors to ensure coverage for sessions while students were on busier clerkships or at clinical sites further from campus. These groups each had a preassigned fourth-year student who had continued in the program after serving as a facilitator during our pilot phase (the prior 6 months). Third- and fourth-year student facilitators signed up to cover sessions in M3/M4 teams based on their availability and, if needed, asked clerkship directors for an excused absence from their own clinical duties to attend the sessions. The Medical Education Office worked with the students to communicate the importance of the learning environment sessions to clerkship directors and coordinators.

In addition to assignments, initial recruitment included a 1-hour mandatory orientation (Appendix B). During this orientation, we explained the overall design of the sessions and reviewed the multiple ways to currently report mistreatment at our institution. During the first 6 months of involvement with the program, third-year student facilitators filled the role of scribe to get acclimated to both the sessions and the clinical setting, while fourth-years served as lead facilitators. At the 6-month mark, the third-year student facilitators attended a 1.5-hour training that marked their transition to acting as lead facilitator, after which fourth-year students transitioned out of the program.

### Midyear Facilitator Training

The goal of our midyear training was to prepare third-year students for their roles as lead facilitators. This entailed providing data and literature pertaining to medical student mistreatment, training on common facilitation techniques, and an approach to documentation. Teaching materials used during the training included a PowerPoint (Appendix C) and a participant packet (Appendix D).

We allocated 1.5 hours for this training but in the future would recommend extending this time to 2–2.5 hours. We wanted to model facilitation techniques as part of the training, and so, we started the session by setting two ground rules: (1) Be actively involved (avoid screens), and (2) information shared in the workshop is not for the purposes of dissemination. We used reflective questions to engage facilitators and encourage a sense of ownership of the program. The group was split up into teams of four for the role-play activity (Appendix E). This 55-minute activity gave each facilitator a chance to practice setting the tone for and facilitating a mock session while peers played the roles of clerkship students. The role-play was loosely based on experiences reported within previous learning environment sessions across several clerkships. At the end of the workshop, we administered a postworkshop survey (Appendix F). The survey used a 5-point Likert scale (0 = *do not agree,* 4 = *highly agree*) and featured a series of statements related to knowledge and attitudes regarding mistreatment, as well as confidence with the role of being a peer-facilitator.

### Learning Environment Sessions

From May 2019 to May 2020, learning environment sessions were held in the six third-year core clerkships at the Boston University School of Medicine. Each session was assigned 1 hour of time for this activity and was staffed by two student leaders involved in the program. One student took meeting minutes while the other served as lead facilitator. In the participant packet (Appendix D), student leaders were provided with a script to introduce the session, a template document for meeting minutes to standardize sessions across clerkships and student leaders, educational materials on mistreatment and barriers to reporting, after-session instructions, and facilitation tips. The scripted introduction outlined the purpose of the sessions, a guarantee of upholding student anonymity, and the way in which the sessions would be conducted. During the session, the scribe filled in a facilitator documentation template that was projected in front of the students. The scribe asked the students to amend phrasing to ensure transparency and agreement on what was being recorded in the minutes. When students spoke about experiences with mistreatment and were willing to identify preceptors, facilitators encouraged them to submit formal reports through our school's anonymous ATM reporting system. Following the session, the minutes were edited for grammar and clarity and submitted to the Medical Education Office and the discipline-specific clerkship director for review. Facilitators were available for follow-up conversations with clerkship directors for any needed clarification. An email was sent at the end of the academic year asking clerkship directors to return any feedback they had about the learning environment sessions.

### Session Minutes Review and Analysis

Within 72 hours of receipt, clerkship directors reviewed the minutes and responded to the Medical Education Office with action steps for any comments about mistreatment or unprofessional behavior in the learning environment, as well as any other areas they wished to respond to. The comments and responses were reviewed by a medical education dean within 72 hours of receipt. Any mistreatment-related comments were forwarded to the chair of the ATM committee and the department chair to monitor trends and action plans to improve student experiences. To protect students, all comments were shared only after clerkship grades had been submitted. Any comments that were concerning for Title IX on review were sent to the Title IX officer by the medical education dean.

## Results

### Student Facilitator Demographics

Twenty-five M3 students were recruited and went through orientation in year 1, and 13 of this group continued as facilitators for a full year. Eleven M3s joined in year 2 and received a 1-hour orientation before starting to scribe during sessions. Ten facilitators attended our midyear training, five over the phone and five in person. In terms of demographic data, student facilitators who attended the workshop ranged from 24 to 30 years in age. We did not collect race or ethnicity data.

### Student Facilitator Feedback

The postworkshop evaluation (Appendix F) had a 100% response rate. The quantitative results are described in the Table. Notably, facilitators highly agreed peer-led sessions were an important avenue for students to process experiences of mistreatment (3.9 out of 4), understood barriers to reporting (3.8 out of 4) and definitions of mistreatment (3.6 out of 4), and felt confident to facilitate these sessions (3.6 out of 4).

**Table.**
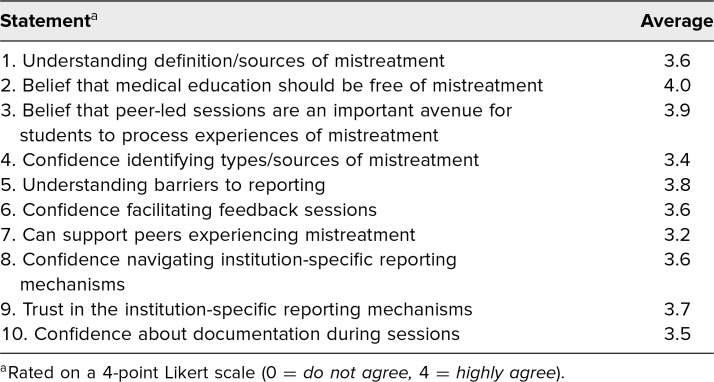
Postworkshop Evaluation Quantitative Data

Open-ended comments from the survey were content-analyzed by authors Martine N. Randolph and Emily Cokorinos Erb looking for large themes. The facilitators reported that their takeaways from the workshop included the following: knowing the differences between mistreatment and unfair treatment, having a framework for defining sources of mistreatment, improved facilitation techniques, and strategies for documentation and communication with the Medical Education Office. Facilitators also reported wanting more time to learn and practice facilitation and more time for role-play activity while getting used to the learning environment sessions script. Suggestions for the next workshop included clarifying the role-play activity and covering how to address difficult comments/situations in the sessions.

Clerkship directors reported that learning environment sessions were a positive addition and a helpful way to obtain real-time feedback about the clerkship. Clerkship director comments for the year included statements that the sessions had become “an invaluable addition to the feedback loop from students… [I] expect it will be a really useful way to better discern how the students are finding the experience” and that the directors had “found [these sessions] so valuable, and I am thrilled to have them continue.”

## Discussion

Addressing medical student mistreatment is an important part of creating a successful learning environment in the clerkship year. Medical students are often ill equipped to identify instances of mistreatment and are hesitant to report these instances. We have found that a peer-led approach is a novel way to encourage reporting of mistreatment and that protected time among peers creates a safe space for clerkship students to talk through real-time scenarios to further understand mistreatment.

Our learning environment sessions provided medical students with an opportunity to learn facilitation skills and showed that students with appropriate training had the confidence to facilitate feedback and support peers experiencing mistreatment. Student facilitators gained a better understanding of mistreatment through the facilitation process, which additionally created a collaboration between students and the medical education administration. This led to real-time action plans by clerkship directors and to changes within the academic year rather than after the year had been completed. We believe other key factors in the success of the learning environment sessions program have been clerkship director support and integration of the sessions into the formal didactic curriculum of the core clerkship year. The time set aside for this activity signaled to the students that the institution was committed to eradicating their mistreatment. This commitment went beyond messaging about a zero-tolerance policy to addressing some of the barriers to reporting that students experienced.

The peer-led model remains vulnerable in several ways, especially without proper guidelines and training for student facilitators. First, student-led discussions have the potential to devolve into unprofessional student behavior during the sessions or into discussions that focus on either nonactionable feedback or feedback outside the scope of the sessions. Training allowed us to standardize the sessions. In our experience, the following types of training are needed: an orientation to the sessions featuring an introductory script and education for the students on the sessions' purpose, as well as setting ground rules for student feedback expectations, and a skills session focused on facilitation skills.

### Limitations

There are several limitations to this program. As yet, the program has not been running long enough to determine its impact on the number of reports submitted about mistreatment or on experiences with mistreatment as measured on the AAMC graduation questionnaire, which could indicate a true change in culture of the learning environment. Our student facilitators were trained to deliver sessions following a standardized script, but they also experimented with ways to get students to talk and reflect. As a result, there may have been some variation in the implementation of these sessions from facilitator to facilitator. Furthermore, the evaluation method for this intervention was a survey of student facilitators' self-reported beliefs, comfort, and confidence in facilitating based on the training. Further study of the effectiveness of the intervention will include mistreatment-reporting behaviors and an evaluation of the graduation questionnaire. Additional limitations include being challenged by fourth-year schedules that included away rotations and travel for residency interviews. As a result, we missed three sessions because of not being able to find coverage or because of errors in scheduling. All student facilitators were able to be dismissed from clinical duties by clerkship directors, who were uniformly supportive of the program. Our in-person sessions ended in March 2020 as a result of the COVID-19 pandemic, yet we continued with virtual sessions via Zoom calls because clerkship directors wanted to seek student feedback regarding the change to virtual learning.

### Next Steps

In the 2020–2021 academic year, fourth-year students have continued to administer the program. With support from the Medical Education Office, student facilitators have been able to pursue medical education and research electives to understand the impact of our program. To evaluate its impact on increasing identification of mistreatment experiences and changing the learning environment, two outcomes will be monitored in the years to come: number of reports submitted to the ATM committee, our formal mechanism for reporting mistreatment, and future AAMC graduation questionnaire data. Next steps also include using a formal assessment tool to survey the students who either attend or facilitate the learning environment sessions as to their perceptions of the benefits they experience as well as the barriers to filing reports they continue to encounter.

## Appendices


Facilitator Application.docxFacilitator Orientation.pptxMidyear Facilitator Training.pptxFacilitator Packet for Midyear Training.docxFacilitator Training Role-Play Activity.docxMidyear Training Evaluation.docx
*All appendices are peer reviewed as integral parts of the Original Publication.*
