# Supplementary material for: Training Medical Students as Peer-Facilitators to Identify Medical Student Mistreatment in the Clerkship Year
Source: MedEdPORTAL. 2021 Sep 27;17:11185. doi: 10.15766/mep_2374-8265.11185 (PMC8473588; doi:10.15766/mep_2374-8265.11185)
Supplement: Supplementary file 1 — Facilitator Application.docxFacilitator Orientation.pptxMidyear Facilitator Training.pptxFacilitator Packet for Midyear Training.docxFacilitator Training Role-Play Activity.docxMidyear Training Evaluation.docx [file mep_2374-8265.11185-s001.zip › C. Midyear Facilitator Training.pptx]

## Slide 1
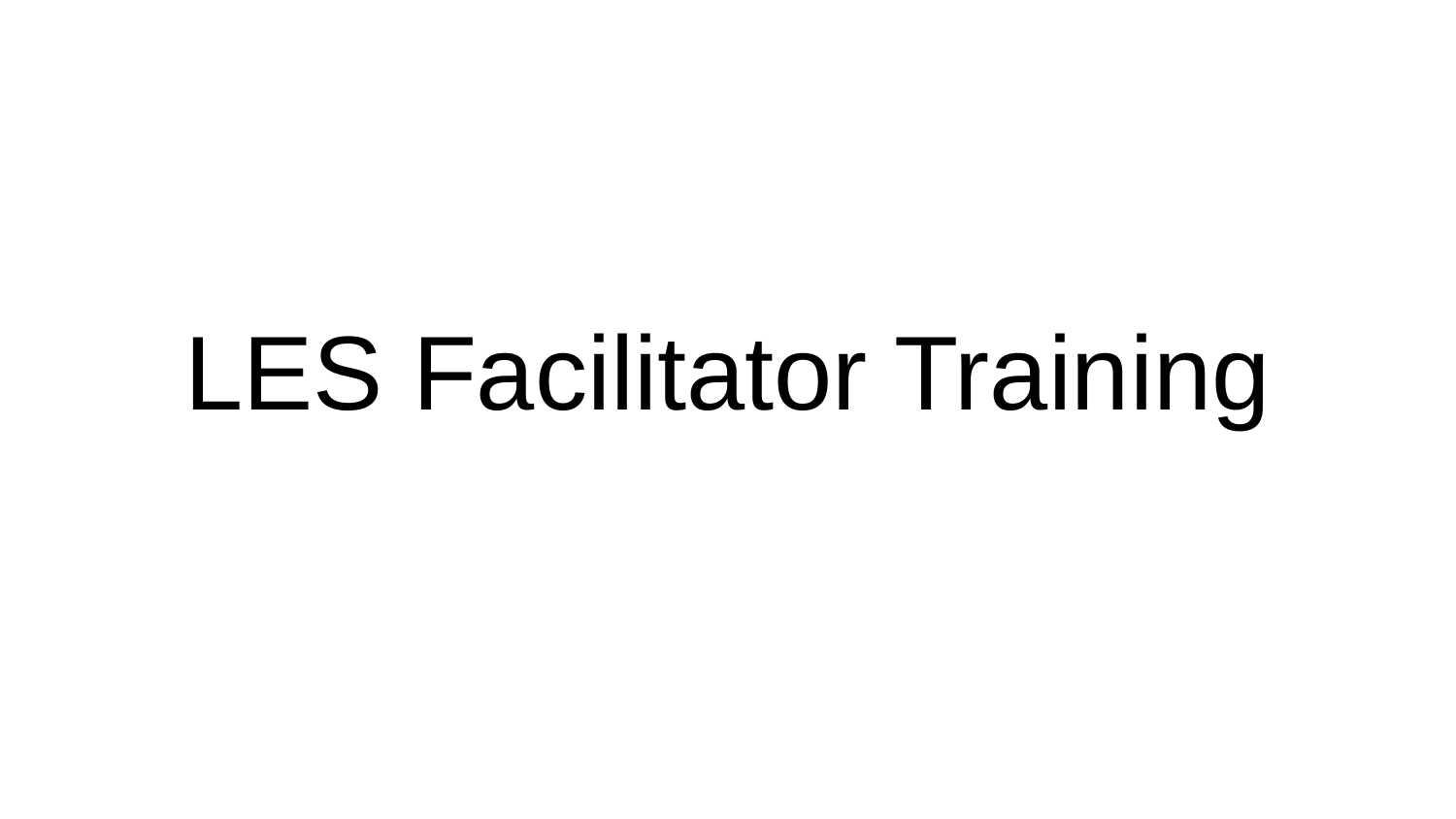

# LES Facilitator Training

## Slide 2
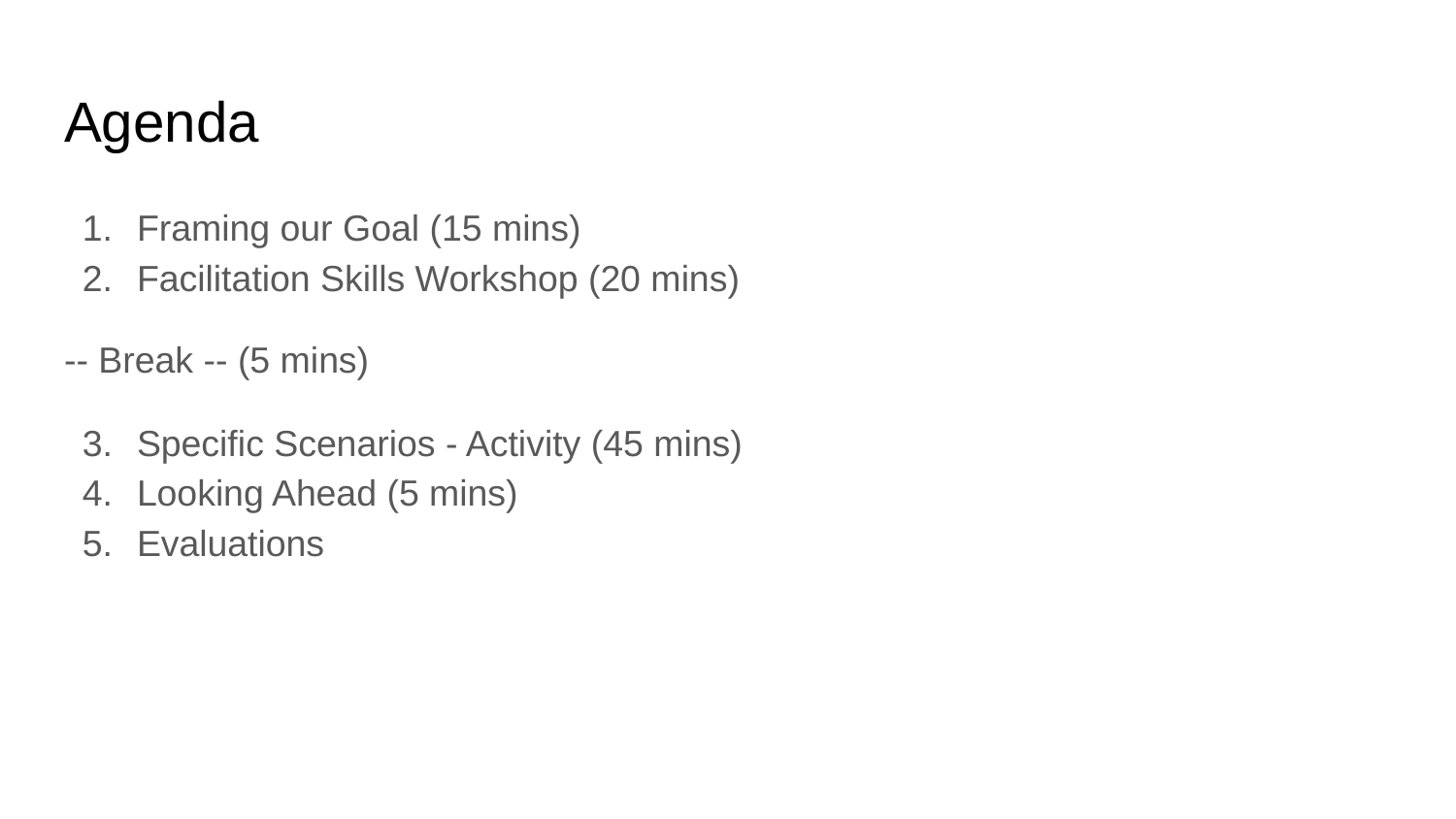

# Agenda
Framing our Goal (15 mins)
Facilitation Skills Workshop (20 mins)
-- Break -- (5 mins)
Specific Scenarios - Activity (45 mins)
Looking Ahead (5 mins)
Evaluations

## Slide 3
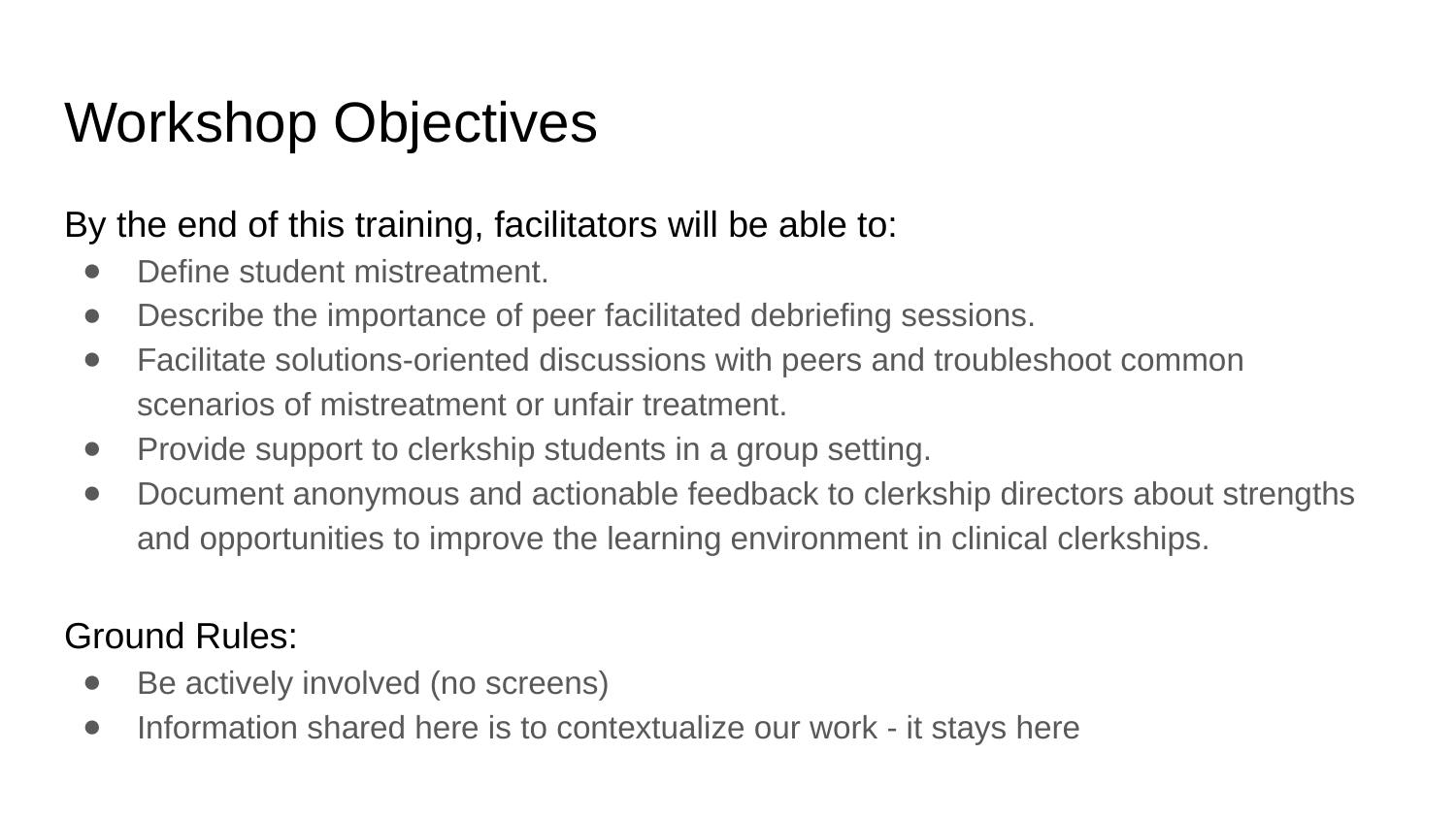

# Workshop Objectives
By the end of this training, facilitators will be able to:
Define student mistreatment.
Describe the importance of peer facilitated debriefing sessions.
Facilitate solutions-oriented discussions with peers and troubleshoot common scenarios of mistreatment or unfair treatment.
Provide support to clerkship students in a group setting.
Document anonymous and actionable feedback to clerkship directors about strengths and opportunities to improve the learning environment in clinical clerkships.
Ground Rules:
Be actively involved (no screens)
Information shared here is to contextualize our work - it stays here

## Slide 4
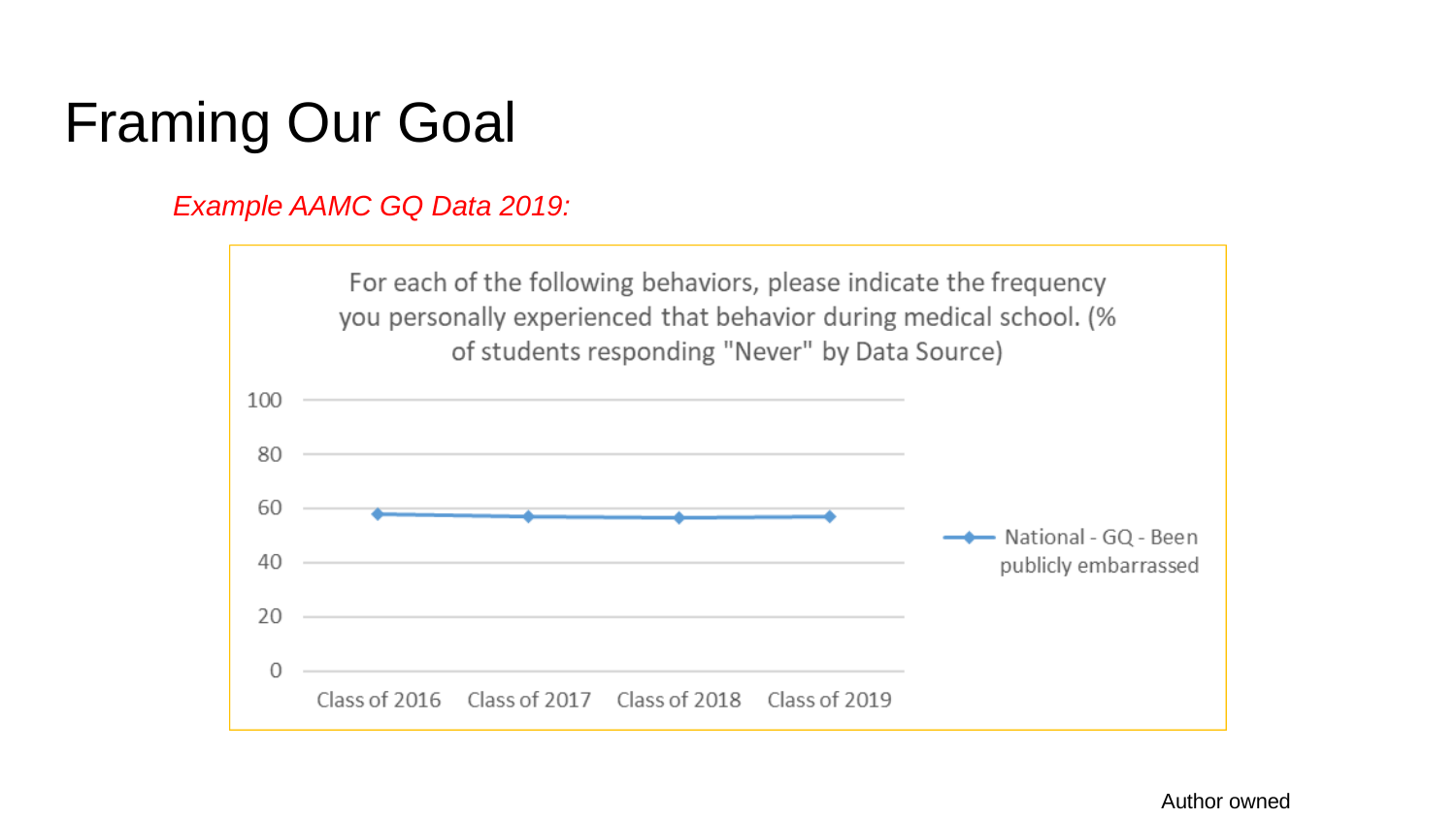

# Framing Our Goal
Example AAMC GQ Data 2019:
Author owned

## Slide 5
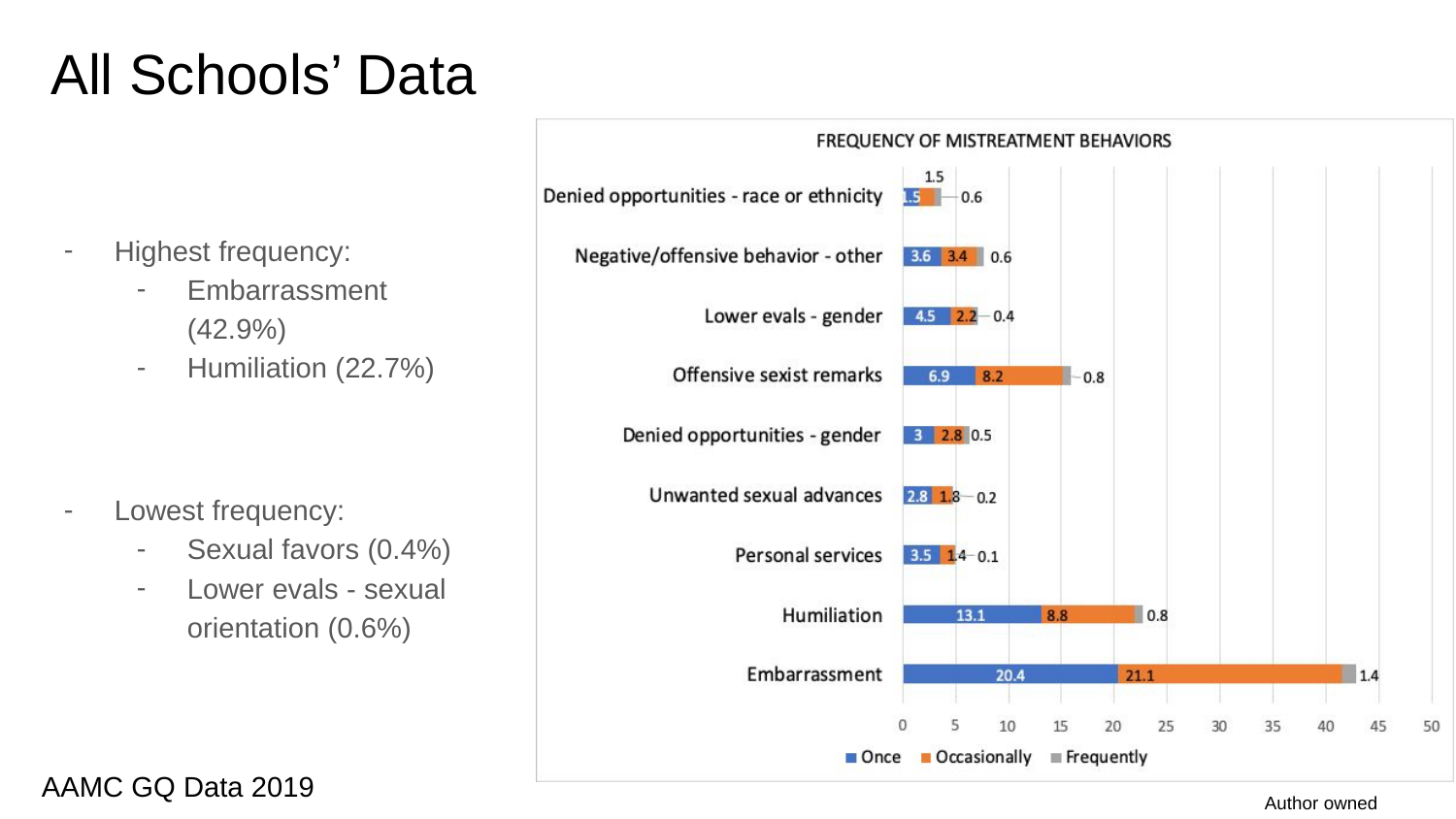

# All Schools’ Data
Highest frequency:
Embarrassment (42.9%)
Humiliation (22.7%)
Lowest frequency:
Sexual favors (0.4%)
Lower evals - sexual orientation (0.6%)
AAMC GQ Data 2019
Author owned

## Slide 6
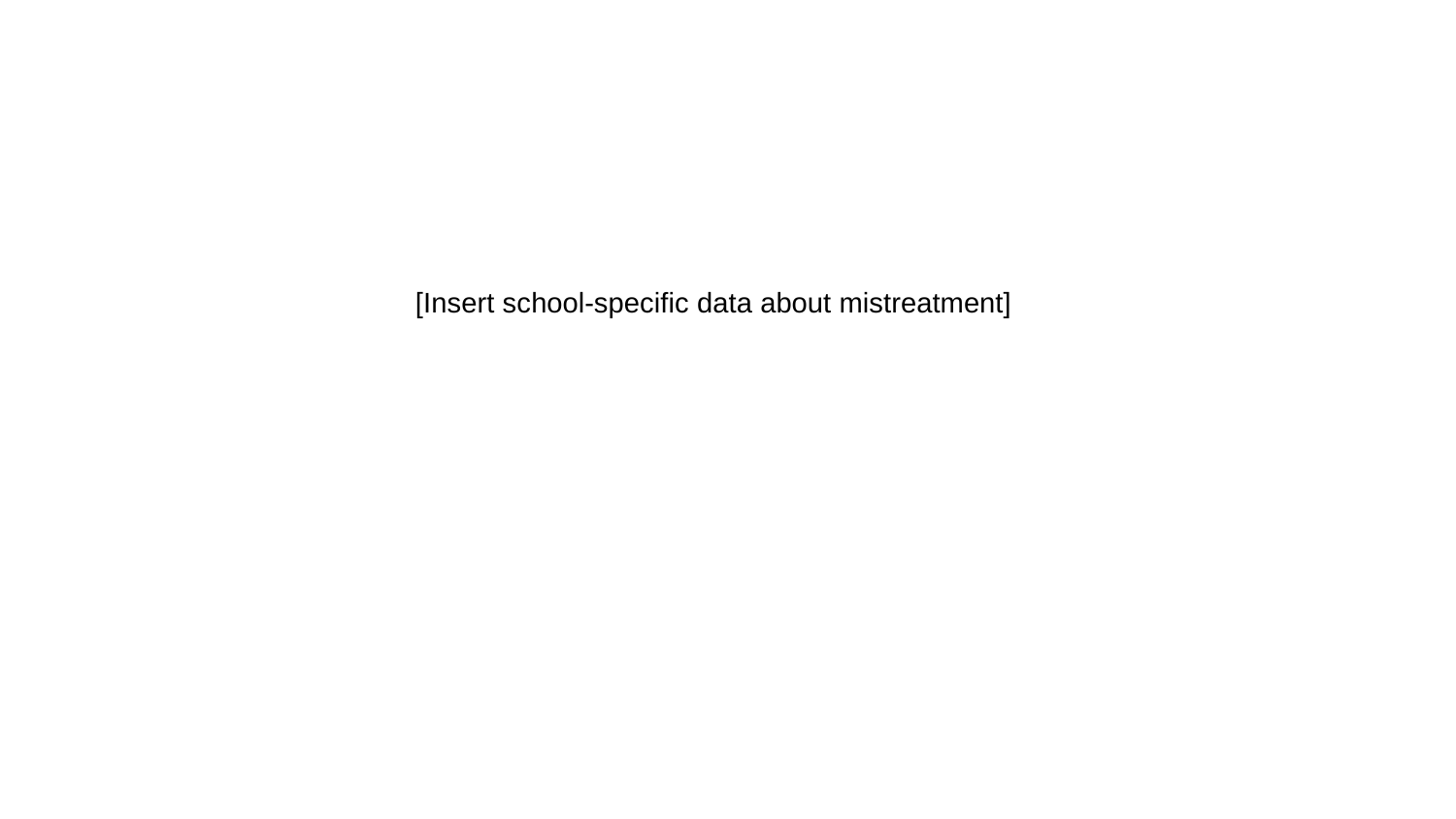

[Insert school-specific data about mistreatment]

## Slide 7
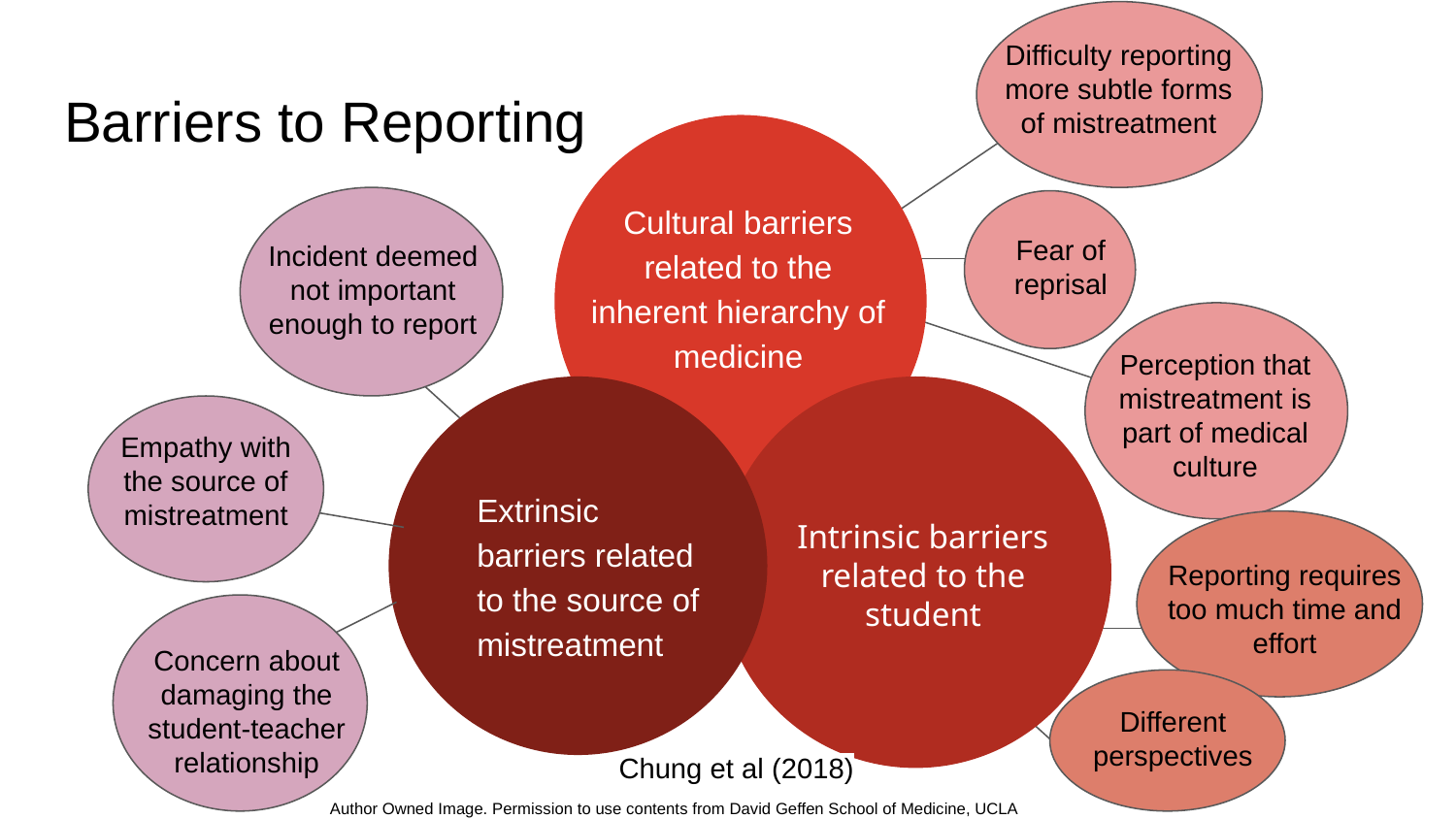

Difficulty reporting more subtle forms of mistreatment
# Barriers to Reporting
Cultural barriers related to the inherent hierarchy of medicine
Fear of reprisal
Incident deemed not important enough to report
Perception that mistreatment is part of medical culture
Extrinsic barriers related to the source of mistreatment
Intrinsic barriers related to the student
Empathy with the source of mistreatment
Reporting requires too much time and effort
Concern about damaging the student-teacher relationship
Different perspectives
Chung et al (2018)
Author Owned Image. Permission to use contents from David Geffen School of Medicine, UCLA

## Slide 8
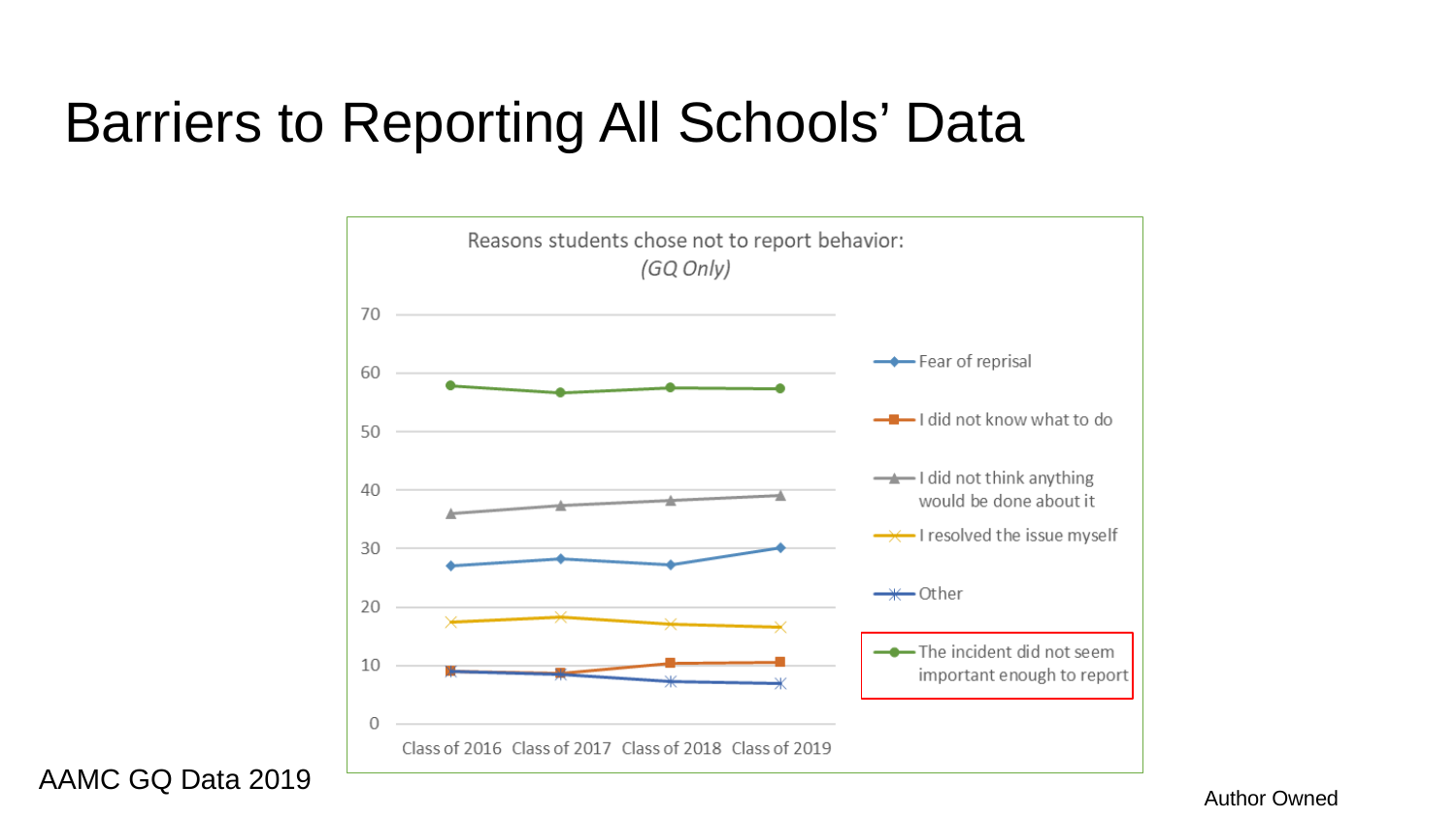

# Barriers to Reporting All Schools’ Data
AAMC GQ Data 2019
Author Owned

## Slide 9
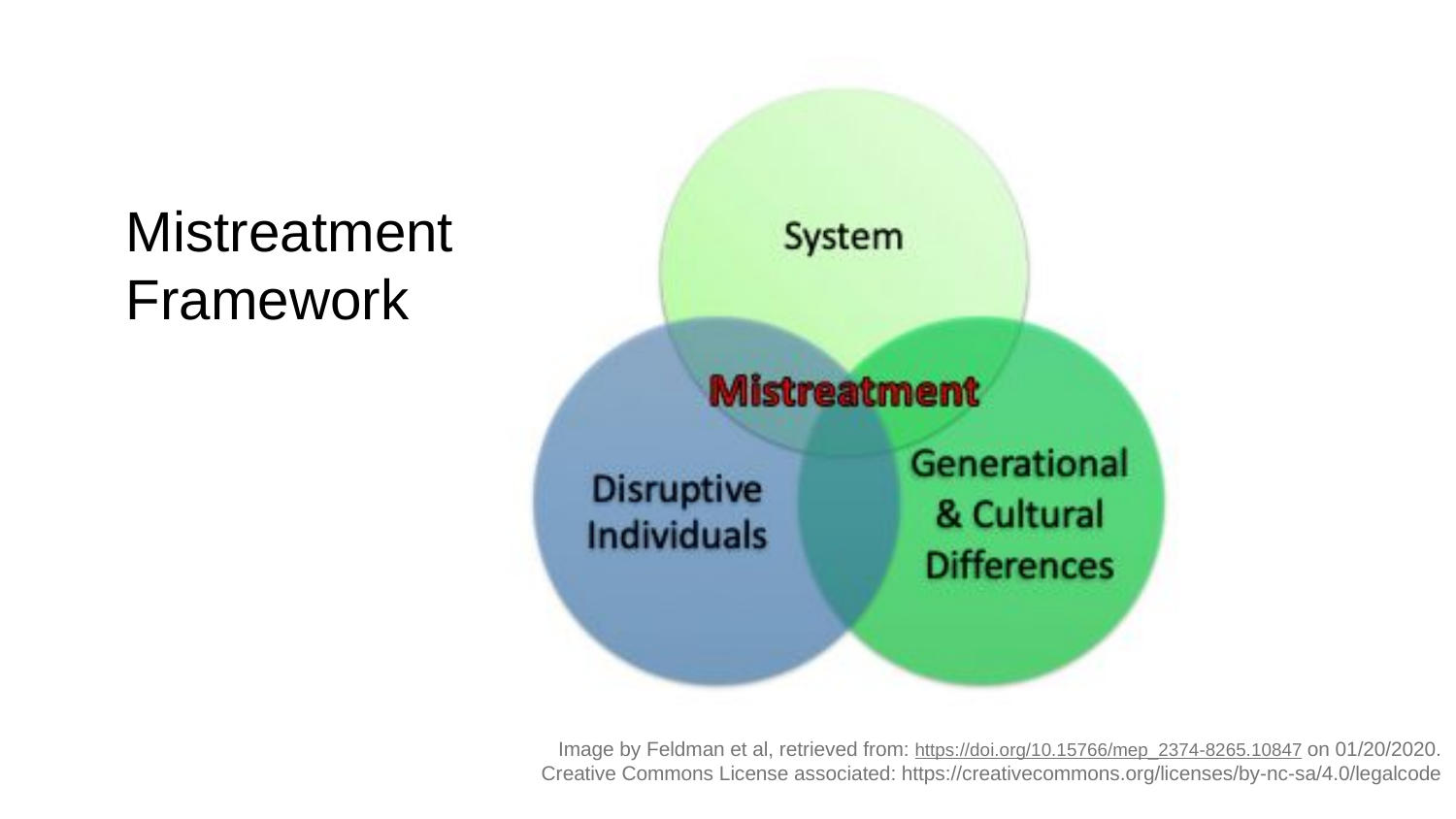

# Mistreatment Framework
Image by Feldman et al, retrieved from: https://doi.org/10.15766/mep_2374-8265.10847 on 01/20/2020. Creative Commons License associated: https://creativecommons.org/licenses/by-nc-sa/4.0/legalcode

## Slide 10
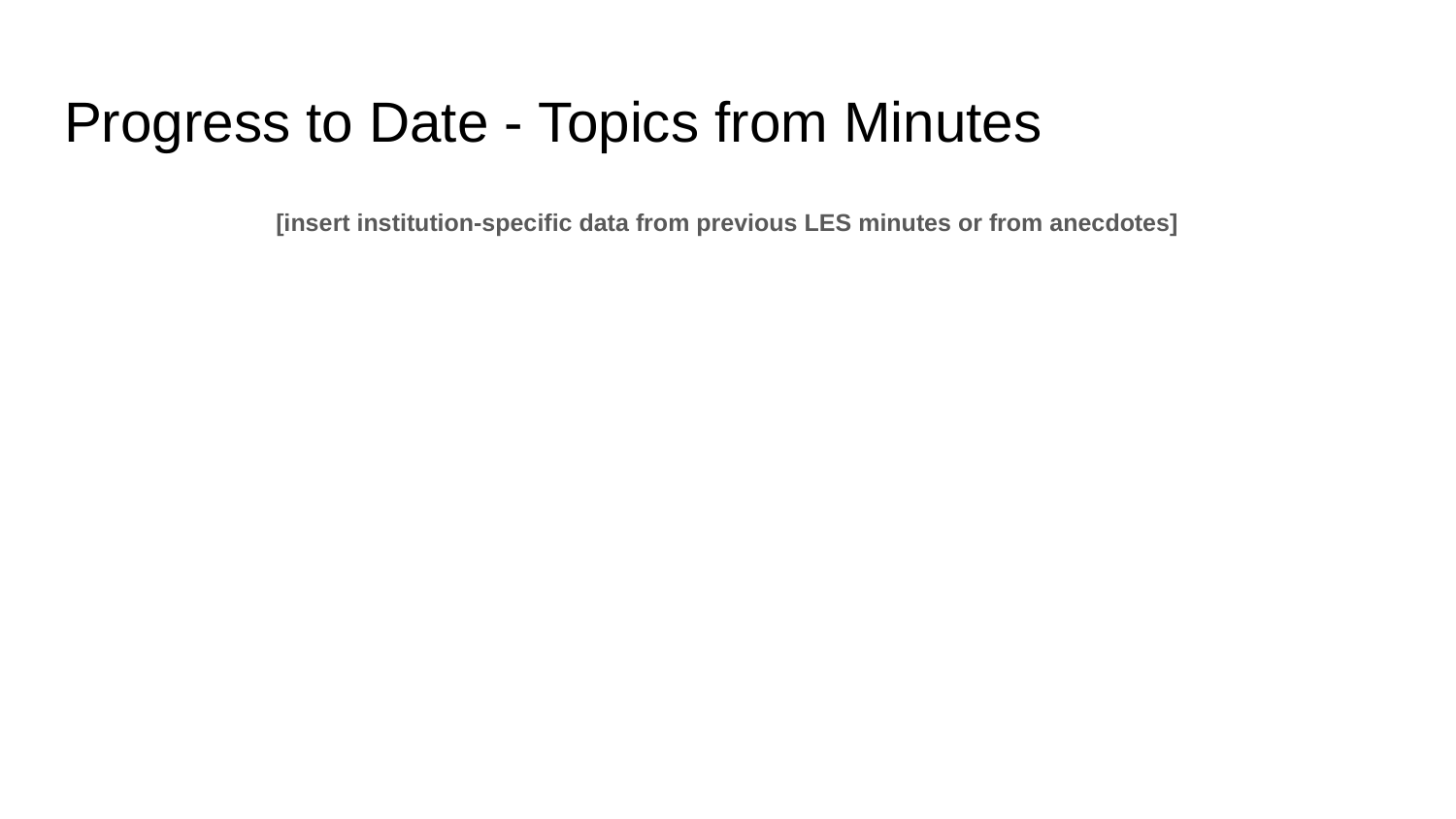

# Progress to Date - Topics from Minutes
[insert institution-specific data from previous LES minutes or from anecdotes]

## Slide 11
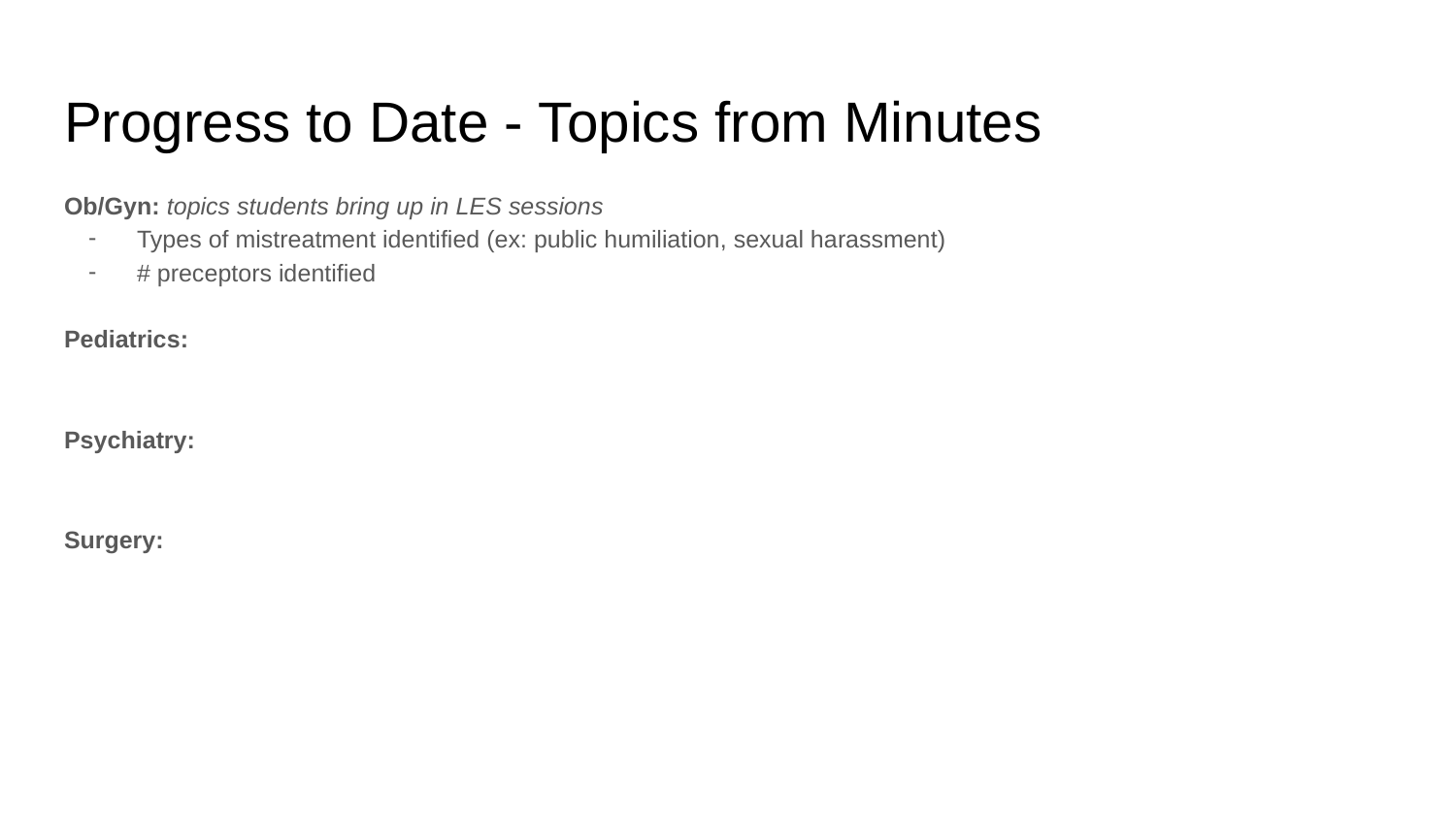

# Progress to Date - Topics from Minutes
Ob/Gyn: topics students bring up in LES sessions
Types of mistreatment identified (ex: public humiliation, sexual harassment)
# preceptors identified
Pediatrics:
Psychiatry:
Surgery:

## Slide 12
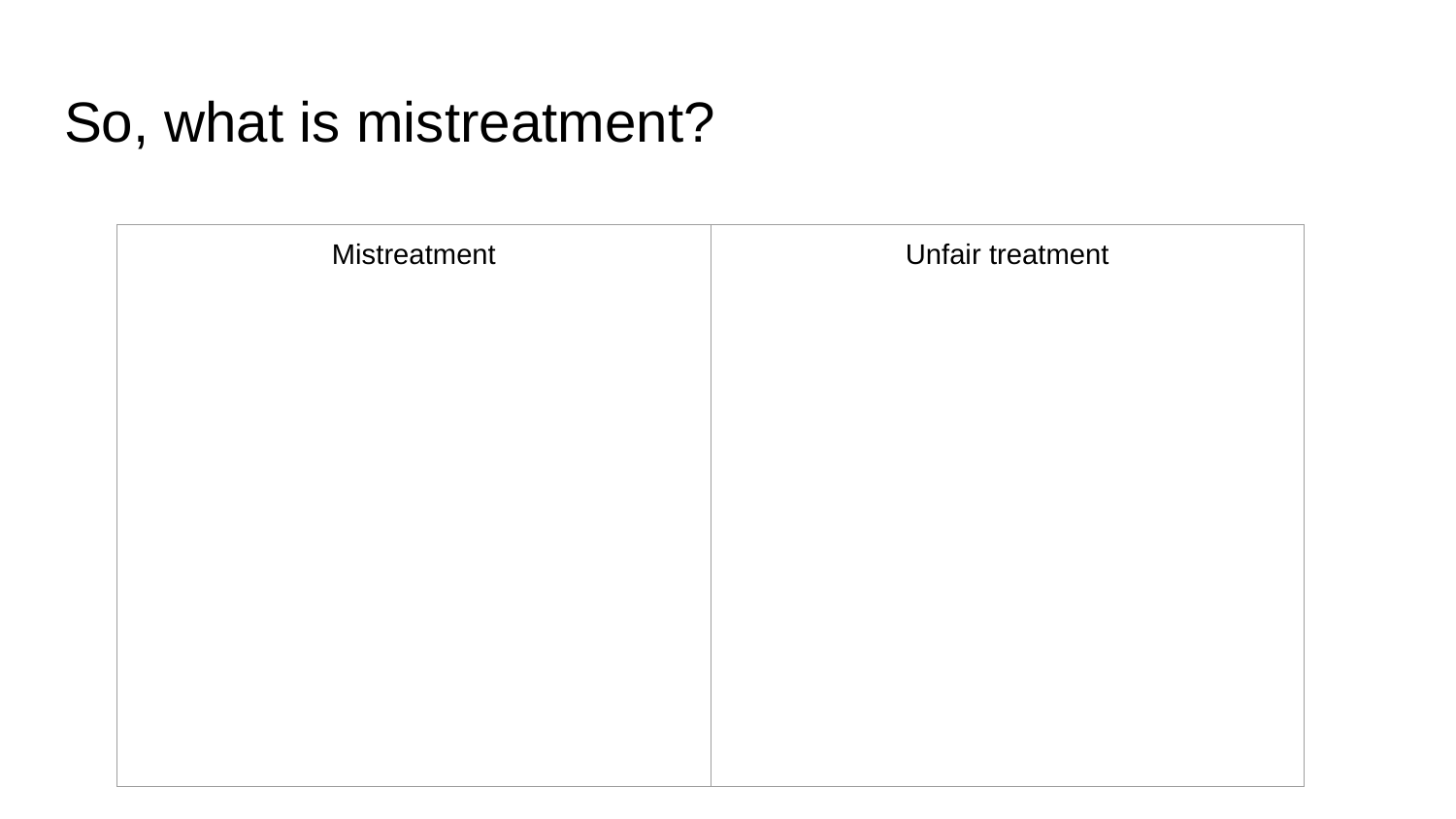

# So, what is mistreatment?
| Mistreatment | Unfair treatment |
| --- | --- |

## Slide 13
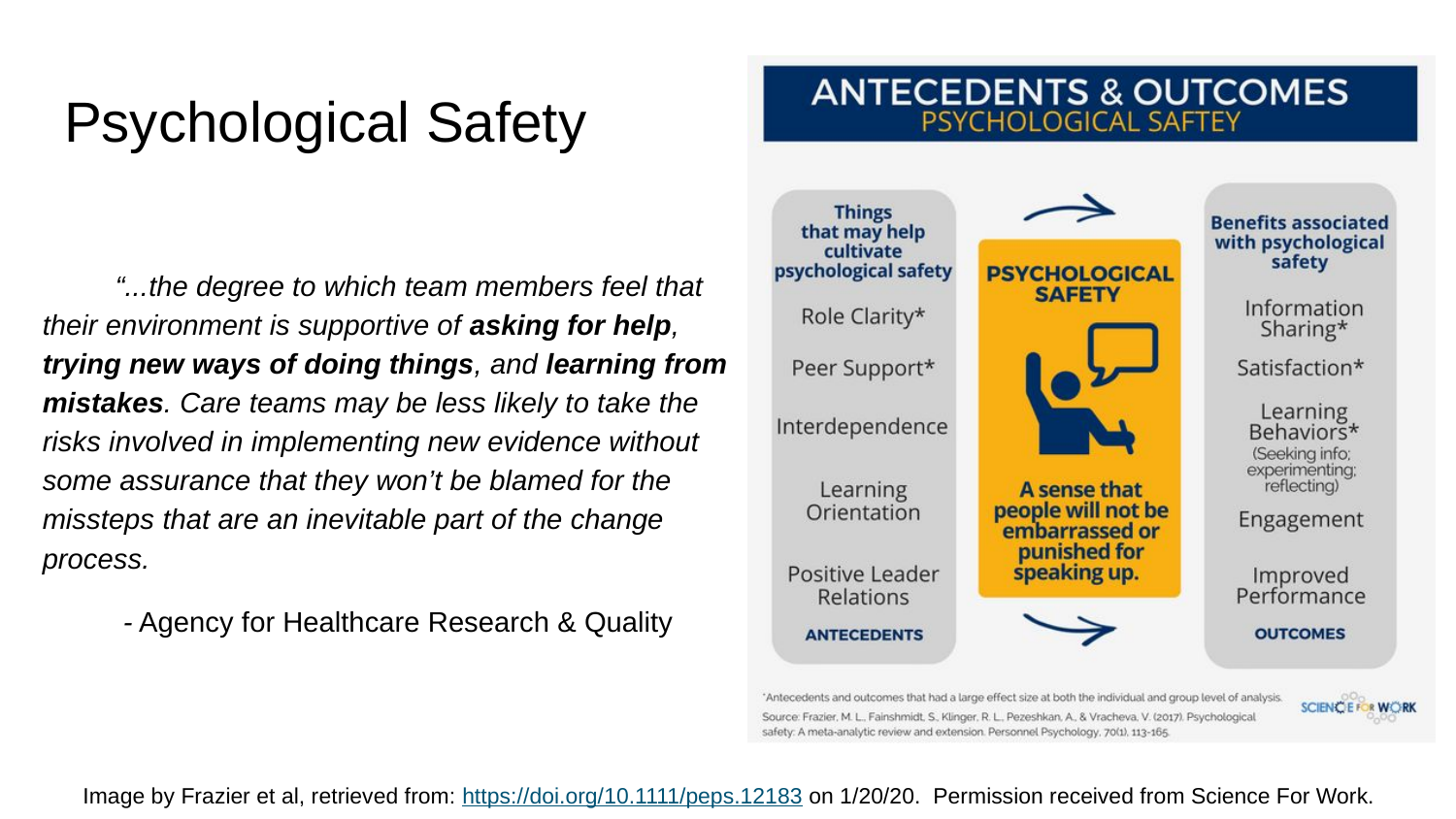

# Psychological Safety
“...the degree to which team members feel that their environment is supportive of asking for help, trying new ways of doing things, and learning from mistakes. Care teams may be less likely to take the risks involved in implementing new evidence without some assurance that they won’t be blamed for the missteps that are an inevitable part of the change process.
 - Agency for Healthcare Research & Quality
Image by Frazier et al, retrieved from: https://doi.org/10.1111/peps.12183 on 1/20/20. Permission received from Science For Work.

## Slide 14
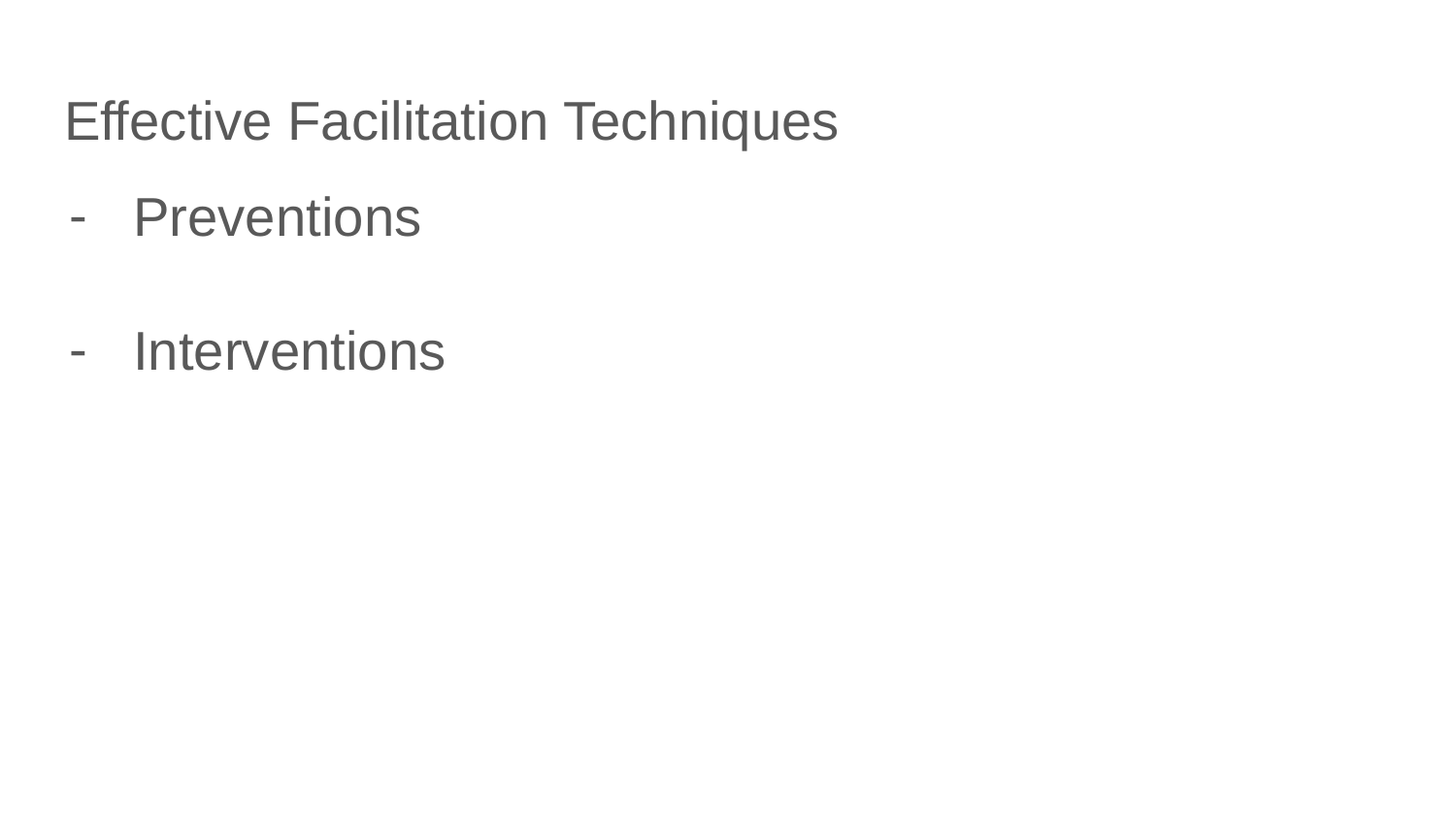

# Effective Facilitation Techniques
Preventions
Interventions

## Slide 15
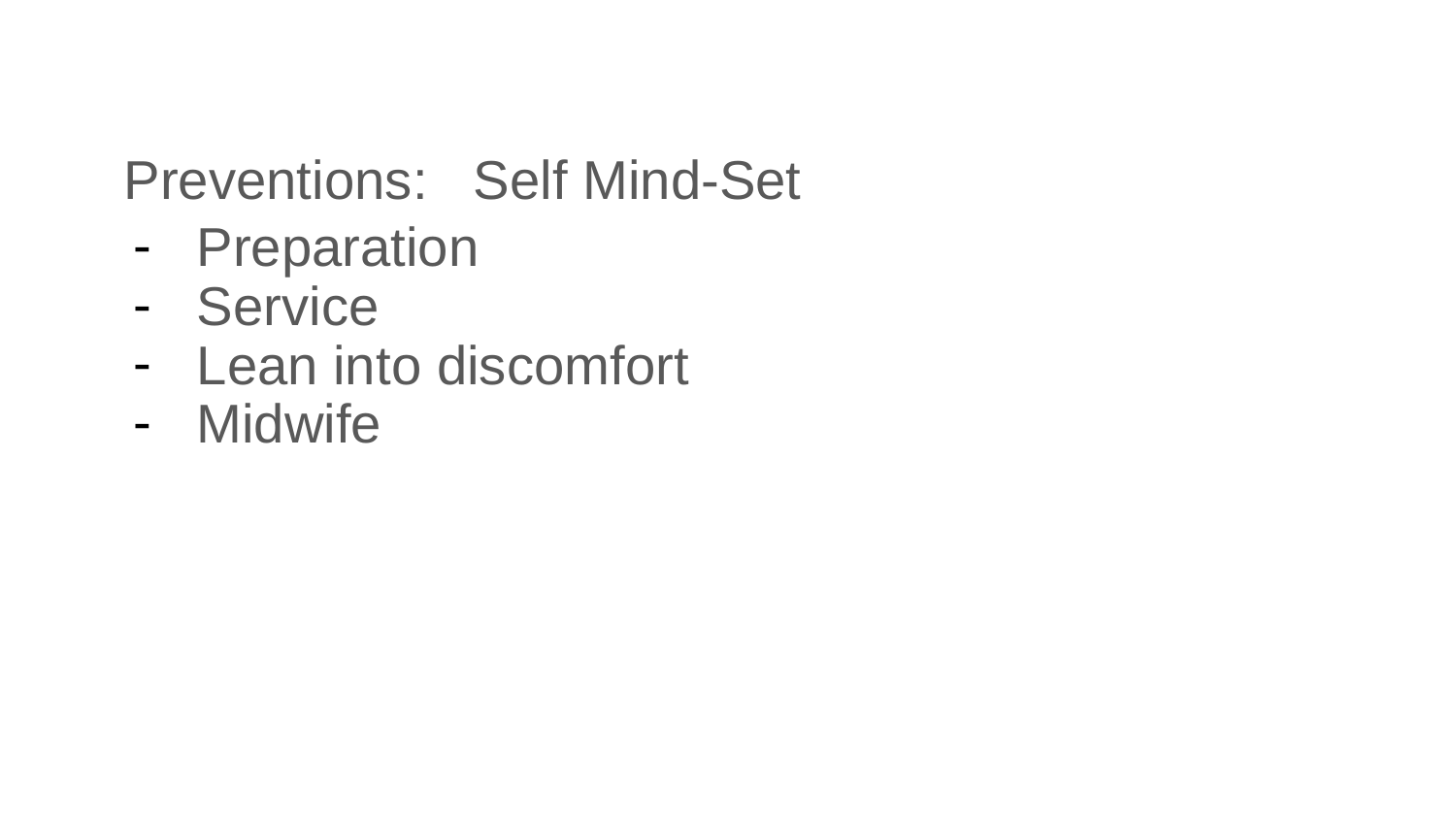

Preventions: Self Mind-Set
Preparation
Service
Lean into discomfort
Midwife

## Slide 16
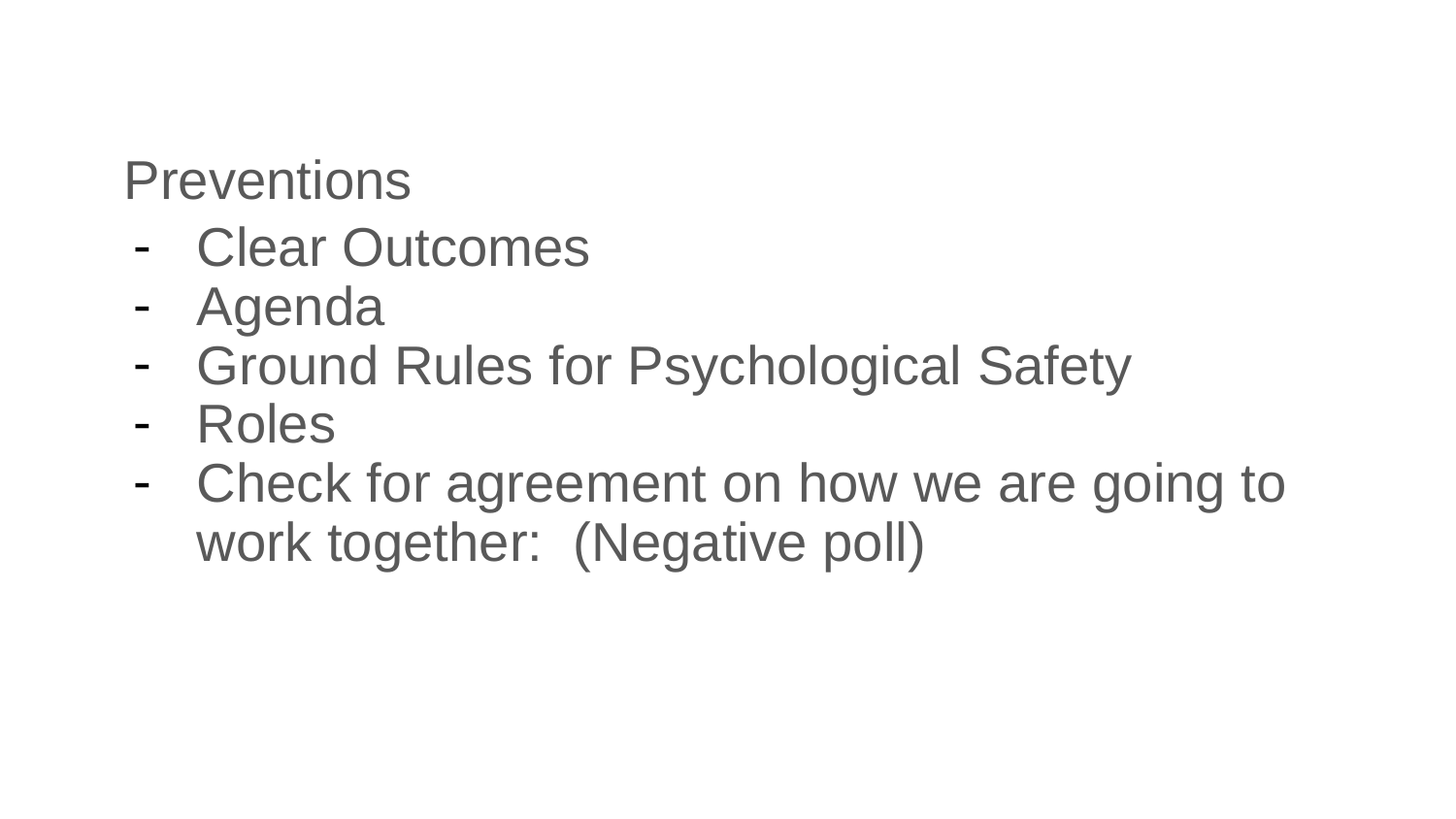

Preventions
Clear Outcomes
Agenda
Ground Rules for Psychological Safety
Roles
Check for agreement on how we are going to work together: (Negative poll)

## Slide 17
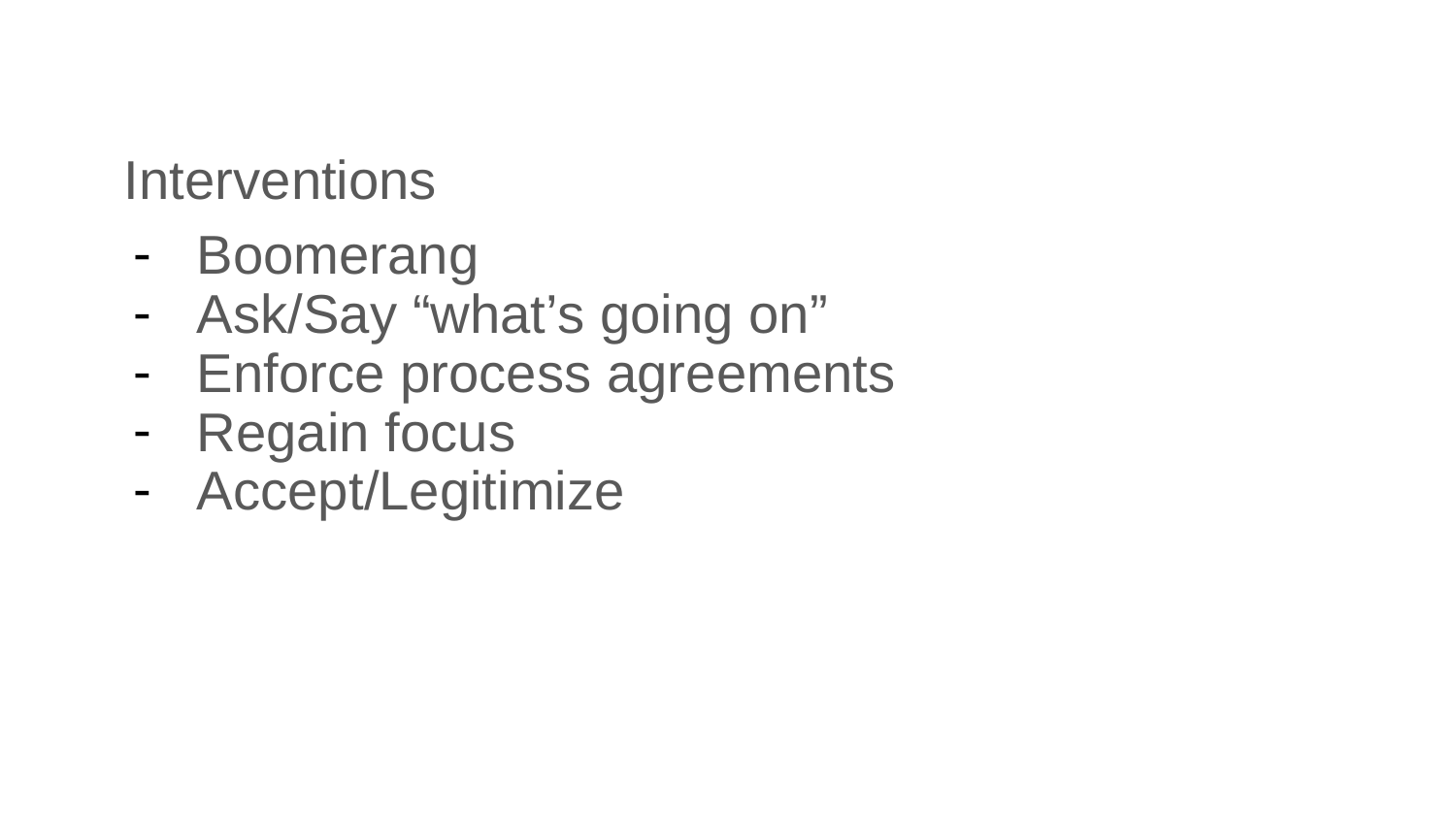

Interventions
Boomerang
Ask/Say “what’s going on”
Enforce process agreements
Regain focus
Accept/Legitimize

## Slide 18
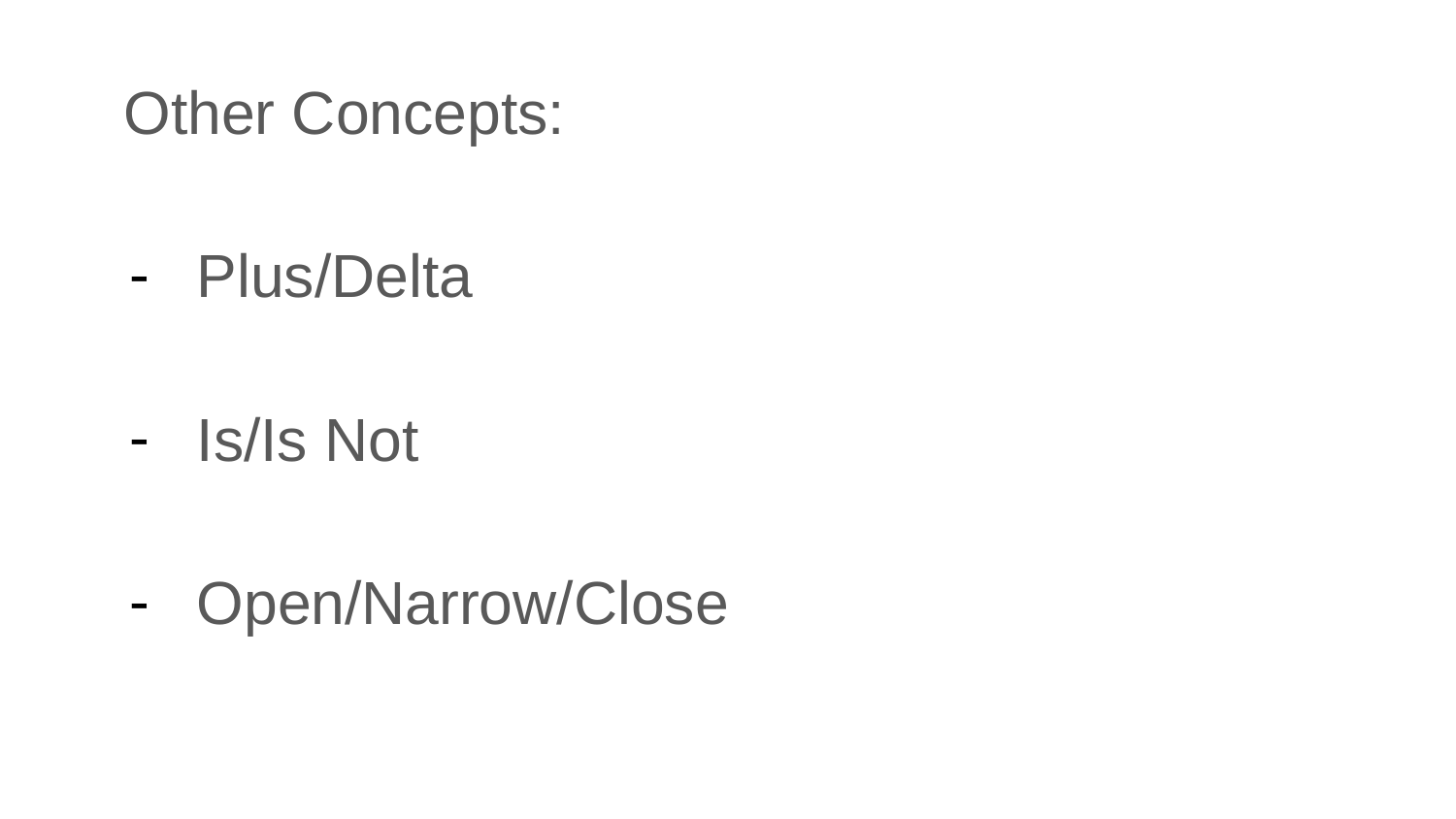

Other Concepts:
Plus/Delta
Is/Is Not
Open/Narrow/Close

## Slide 19
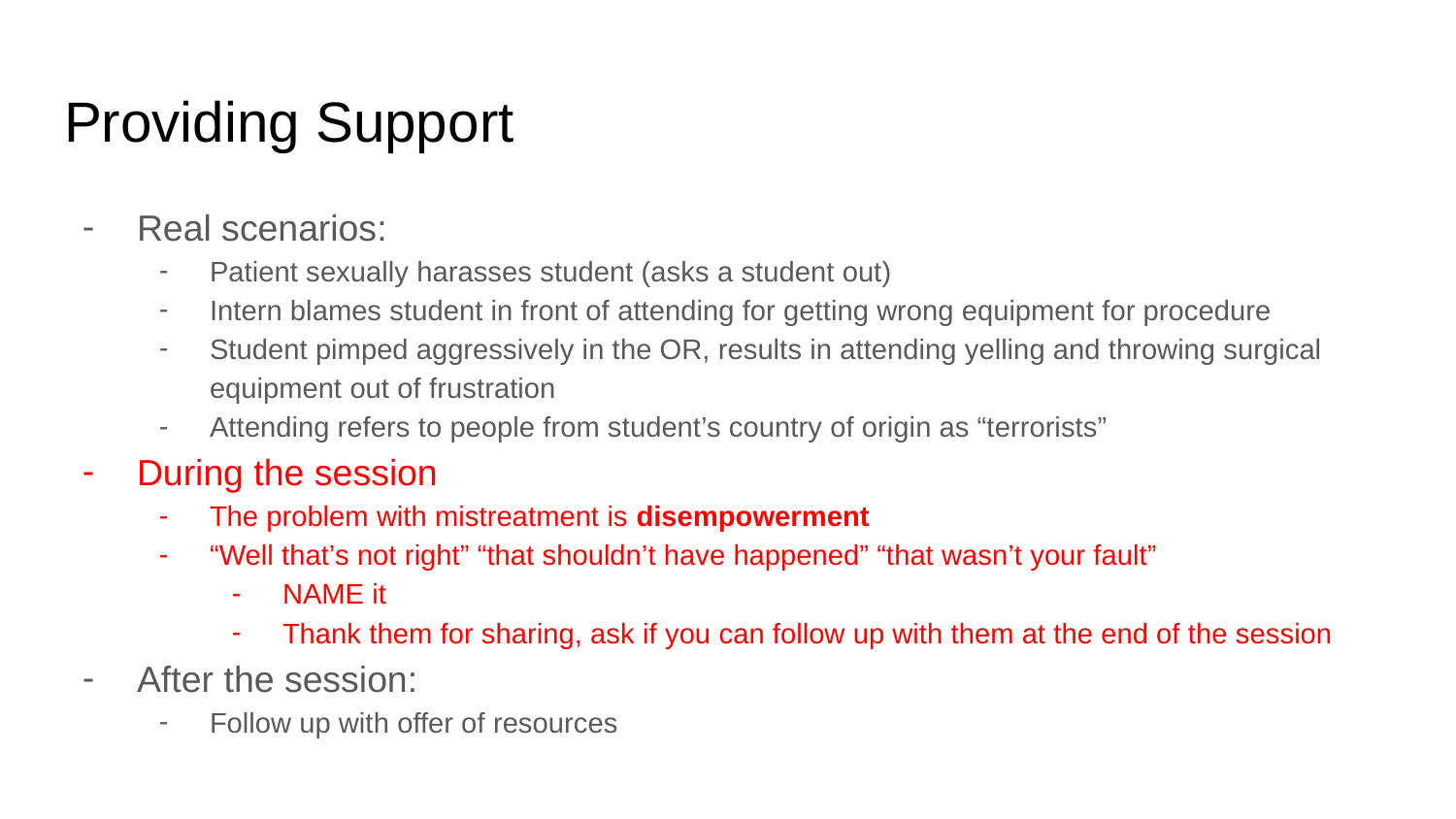

# Providing Support
Real scenarios:
Patient sexually harasses student (asks a student out)
Intern blames student in front of attending for getting wrong equipment for procedure
Student pimped aggressively in the OR, results in attending yelling and throwing surgical equipment out of frustration
Attending refers to people from student’s country of origin as “terrorists”
During the session
The problem with mistreatment is disempowerment
“Well that’s not right” “that shouldn’t have happened” “that wasn’t your fault”
NAME it
Thank them for sharing, ask if you can follow up with them at the end of the session
After the session:
Follow up with offer of resources

## Slide 20
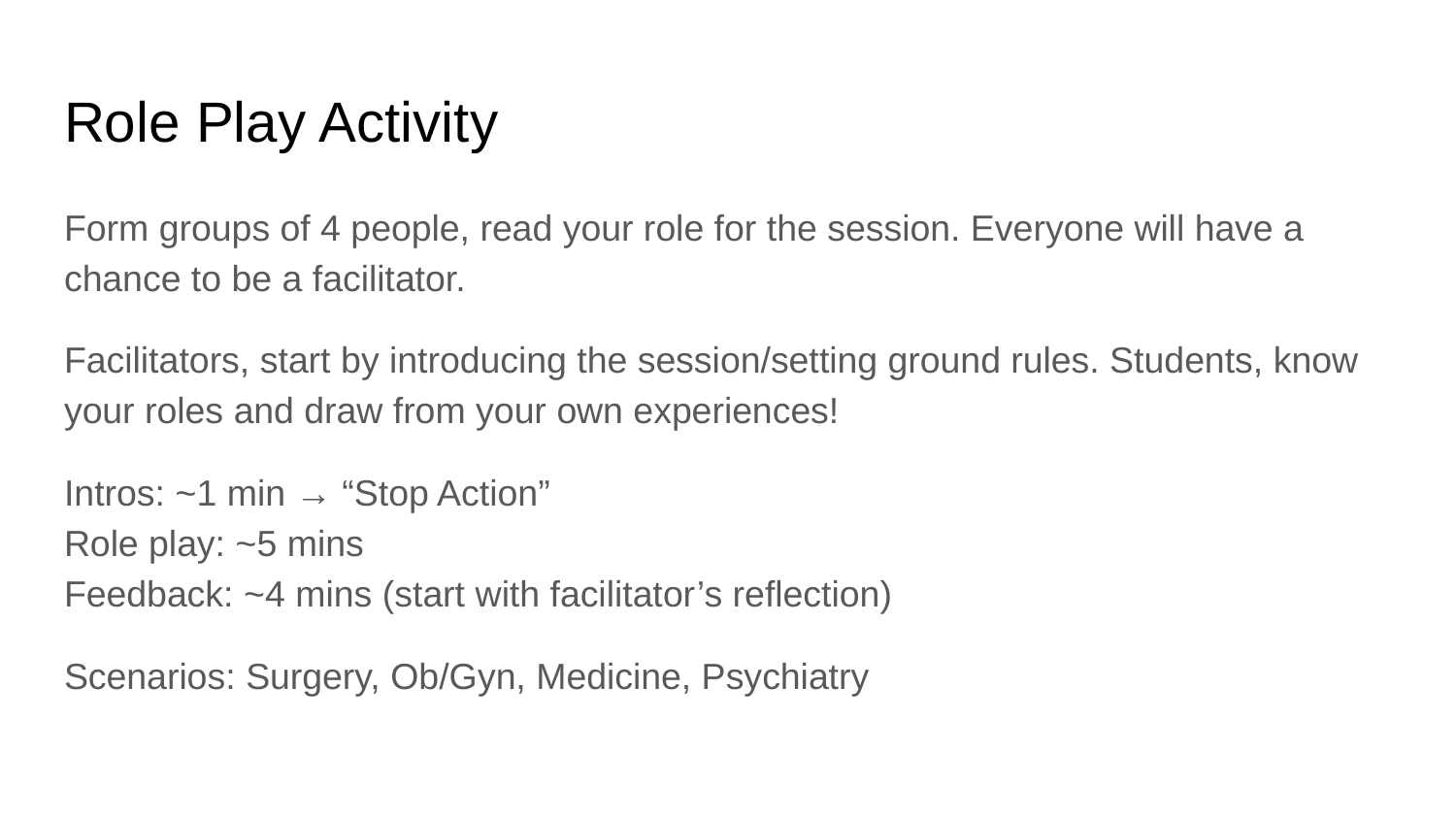

# Role Play Activity
Form groups of 4 people, read your role for the session. Everyone will have a chance to be a facilitator.
Facilitators, start by introducing the session/setting ground rules. Students, know your roles and draw from your own experiences!
Intros: ~1 min → “Stop Action”
Role play: ~5 mins
Feedback: ~4 mins (start with facilitator’s reflection)
Scenarios: Surgery, Ob/Gyn, Medicine, Psychiatry

## Slide 21
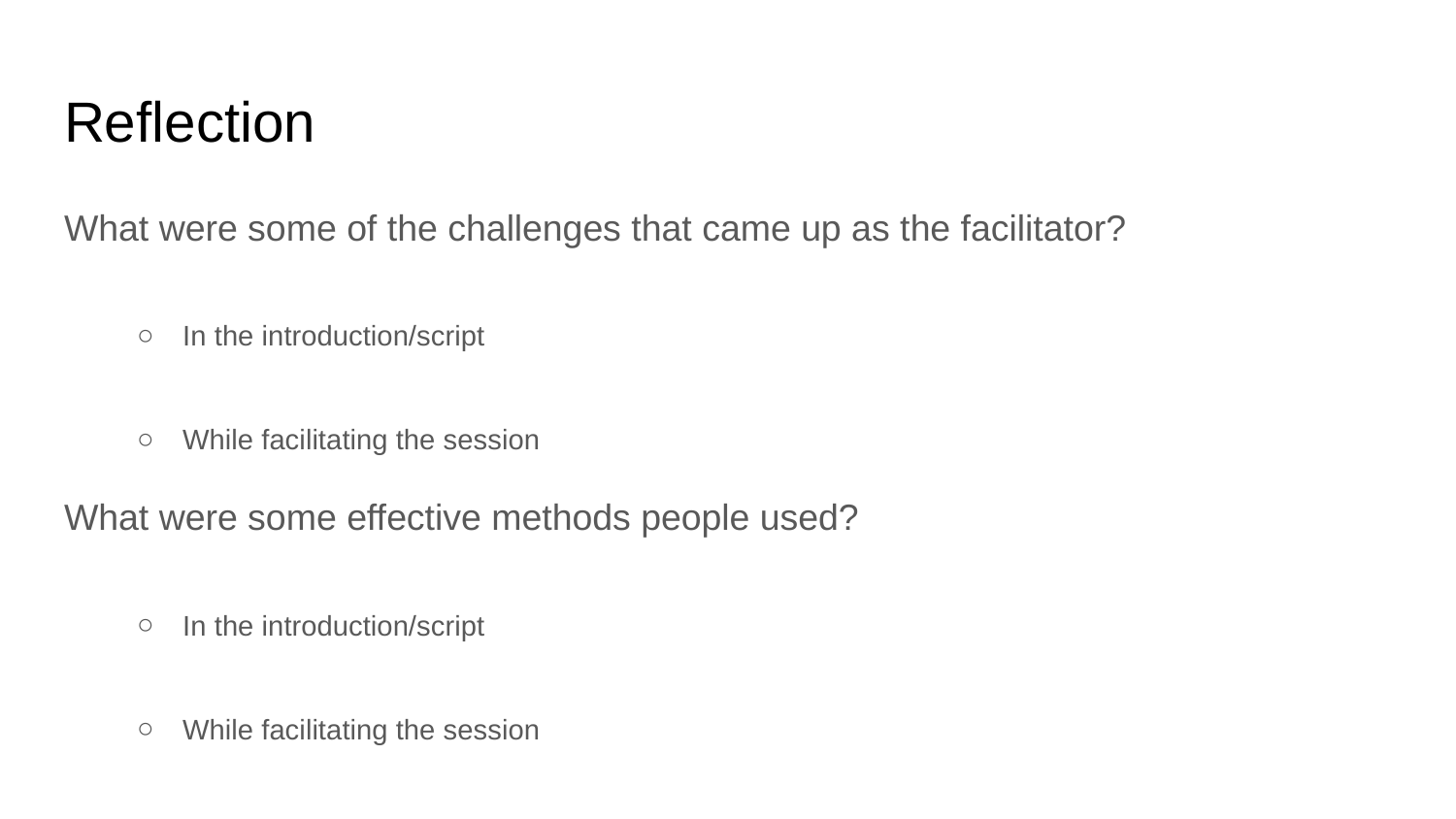

# Reflection
What were some of the challenges that came up as the facilitator?
In the introduction/script
While facilitating the session
What were some effective methods people used?
In the introduction/script
While facilitating the session

## Slide 22
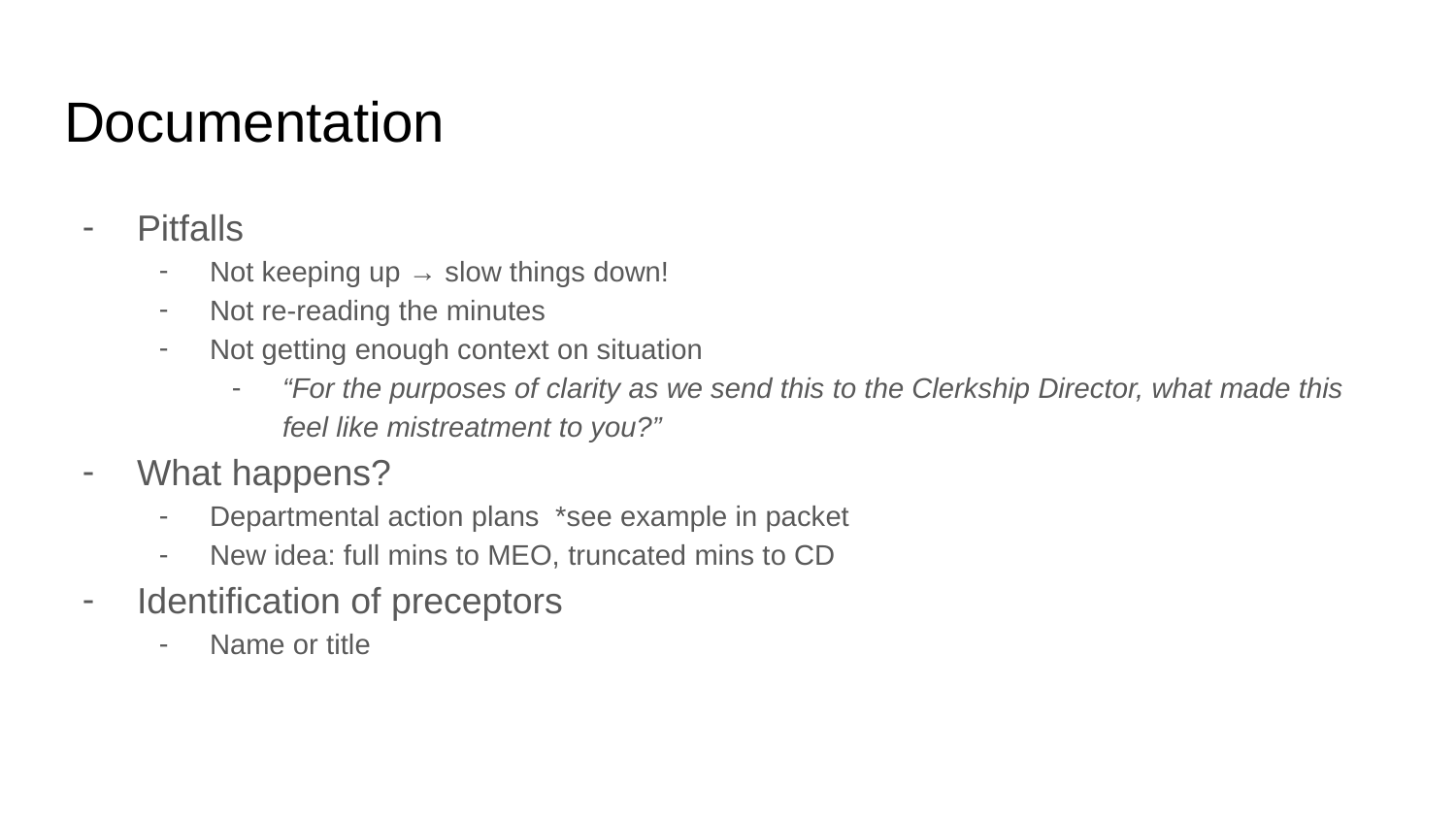

# Documentation
Pitfalls
Not keeping up → slow things down!
Not re-reading the minutes
Not getting enough context on situation
“For the purposes of clarity as we send this to the Clerkship Director, what made this feel like mistreatment to you?”
What happens?
Departmental action plans *see example in packet
New idea: full mins to MEO, truncated mins to CD
Identification of preceptors
Name or title

## Slide 23
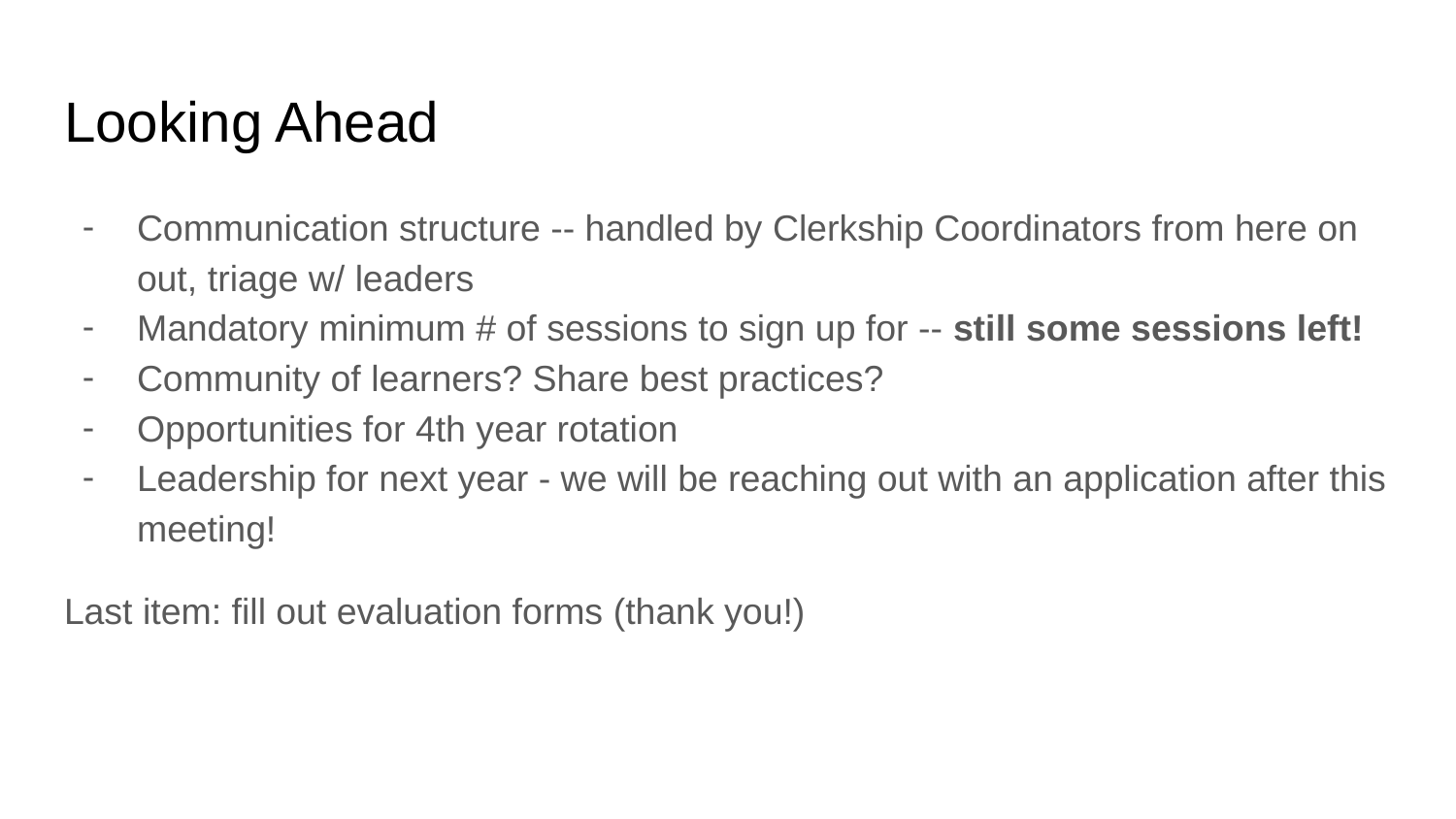

# Looking Ahead
Communication structure -- handled by Clerkship Coordinators from here on out, triage w/ leaders
Mandatory minimum # of sessions to sign up for -- still some sessions left!
Community of learners? Share best practices?
Opportunities for 4th year rotation
Leadership for next year - we will be reaching out with an application after this meeting!
Last item: fill out evaluation forms (thank you!)

## Slide 24
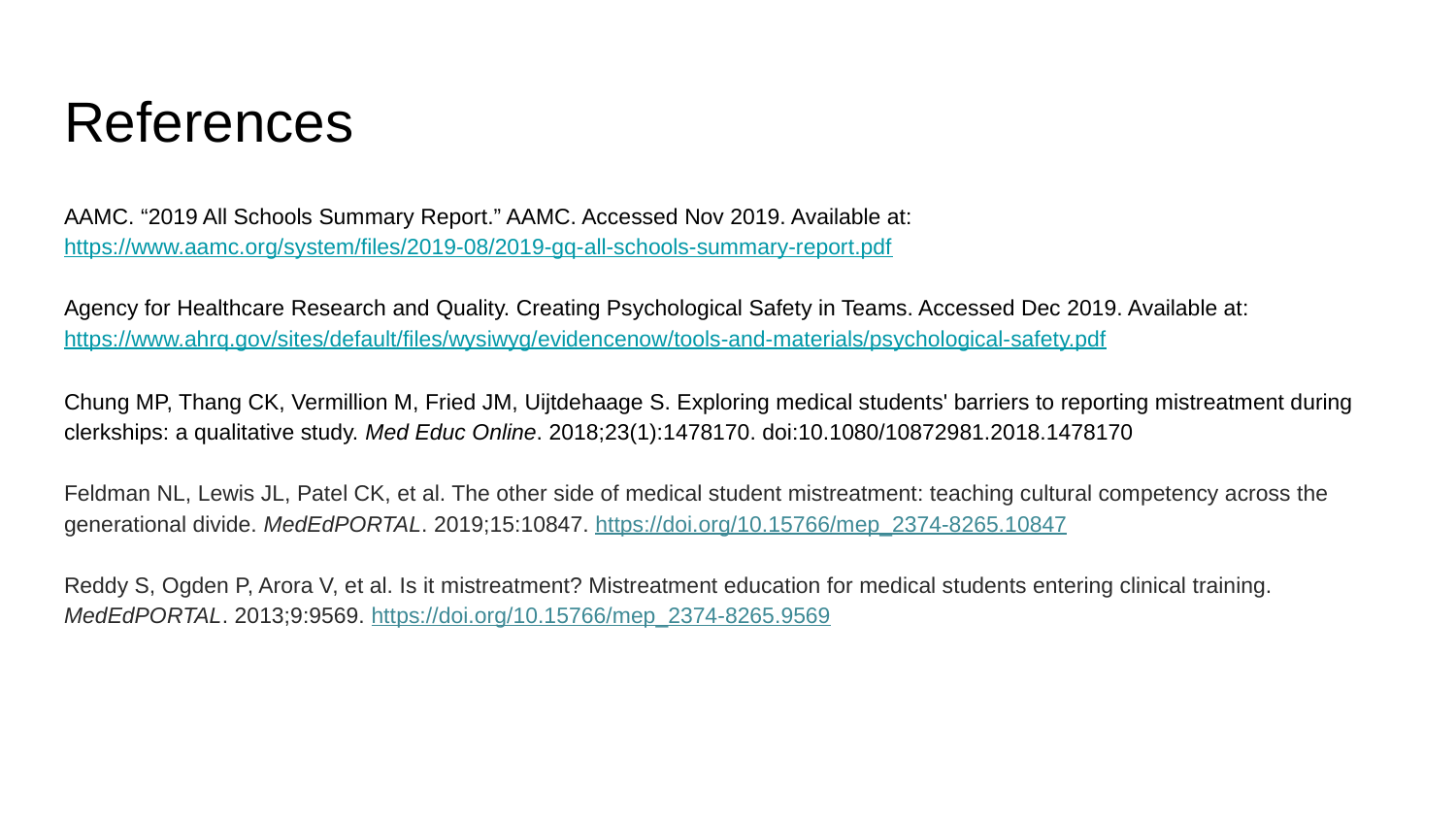

# References
AAMC. “2019 All Schools Summary Report.” AAMC. Accessed Nov 2019. Available at: https://www.aamc.org/system/files/2019-08/2019-gq-all-schools-summary-report.pdf
Agency for Healthcare Research and Quality. Creating Psychological Safety in Teams. Accessed Dec 2019. Available at: https://www.ahrq.gov/sites/default/files/wysiwyg/evidencenow/tools-and-materials/psychological-safety.pdf
Chung MP, Thang CK, Vermillion M, Fried JM, Uijtdehaage S. Exploring medical students' barriers to reporting mistreatment during clerkships: a qualitative study. Med Educ Online. 2018;23(1):1478170. doi:10.1080/10872981.2018.1478170
Feldman NL, Lewis JL, Patel CK, et al. The other side of medical student mistreatment: teaching cultural competency across the generational divide. MedEdPORTAL. 2019;15:10847. https://doi.org/10.15766/mep_2374-8265.10847
Reddy S, Ogden P, Arora V, et al. Is it mistreatment? Mistreatment education for medical students entering clinical training. MedEdPORTAL. 2013;9:9569. https://doi.org/10.15766/mep_2374-8265.9569
