# Supplementary material for: Training Medical Students as Peer-Facilitators to Identify Medical Student Mistreatment in the Clerkship Year
Source: MedEdPORTAL. 2021 Sep 27;17:11185. doi: 10.15766/mep_2374-8265.11185 (PMC8473588; doi:10.15766/mep_2374-8265.11185)
Supplement: Supplementary file 1 — Facilitator Application.docxFacilitator Orientation.pptxMidyear Facilitator Training.pptxFacilitator Packet for Midyear Training.docxFacilitator Training Role-Play Activity.docxMidyear Training Evaluation.docx [file mep_2374-8265.11185-s001.zip › D. Facilitator Packet for Midyear Training.docx]

**Learning Environment Sessions**

FACILITATOR TRAINING

Contents

1. Barriers to Reporting
2. What is mistreatment? MISTREAT Pocket Card
3. Learning Environment Session Group Facilitation Script Template
4. Learning Environment Session Facilitator Documentation Template
5. After Session Instruction
6. Facilitation Tips
7. References

**Exploring medical students' barriers to reporting mistreatment during clerkships: a qualitative study**

Source: Chung MP, Thang CK, Vermillion M, Fried JM, Uijtdehaage S. Exploring medical students' barriers to reporting mistreatment during clerkships: a qualitative study. *Med Educ Online*. 2018;23(1):1478170. DOI: [10.1080/10872981.2018.1478170](https://doi.org/10.1080/10872981.2018.1478170)

**Fear of Reprisal**

*Fear of retribution is probably the greatest barrier on everyone’s mind. Obviously if you’re reporting it*

*within the year that you’re doing your clerkship, it can be easily traced back to you because the incident*

*is so fresh. The community is so small. Anyone who speaks up is labeled as a whistleblower. – MS4*

**Perception that mistreatment is part of medical culture**

*Students ‘learned their place’ in occupying the lowest tier of the medical hierarchy, citing the power differential as a major reason as to why they were especially vulnerable to mistreatment by residents and attending physicians. One student called medical school a ‘hazing process,’ and had come to accept mistreatment because ‘that’s just the way it is.’ To cope with this learned powerlessness, many students adopted the belief that they needed to develop resilience in order to excel on clinical clerkships. Multiple students in one focus group nodded in agreement upon hearing the following comment from a student: When it comes to me, I didn’t report most things because I’m like, ‘I need to man up.’ – MS4*

**Concern about damaging the student-teacher relationship**

*I can only imagine how much attendings hate it when medical students don’t open honest dialogue with them and just destroy them in the final evaluation. The worst part of reporting is that nobody wins.*

*– MS4*

**Empathy with the source of mistreatment**

*We seem to imagine ourselves as pinnacles of equality, compassion, and moral justice. We forget that physicians are, at the end of the day, just people. Just like your next-door neighbor, people in the medical*

*profession are just as privy to fatigue, worries, and stress. And this stress manifests in many different*

*ways. – MS4*

**Incident deemed not important enough to report**

*I claimed that I had never experienced mistreatment before. That’s after having been called North Korean, being confused with other Asians, being told I look like a Japanese baby. So why do I not count myself as mistreated? Because I just don’t care. It’s only mistreatment if you let yourself be affected. – MS4*

**Reporting process takes too much time and effort**

*For a lot of the mistreatment it’s small enough where reporting it is too much work. I have to describe it in*

*detail, I’d rather just deal with it. I’m tired, I’d rather go sleep or study for my shelf exam. – MS3*

**Differing perspectives**

*The downside is that everyone thinks we’re too coddled. They joke about what they can or can’t do to medical students. They’re aware of the issue but in a bad way. They think we’re overly sensitive. – MS4*

**MISTREAT Pocket Card**

Source: Reddy S, Ogden P, Arora V, et al. Is it mistreatment? Mistreatment education for medical students entering clinical training. MedEdPORTAL. 2013;9:9569. <https://doi.org/10.15766/mep_2374-8265.9569>

*
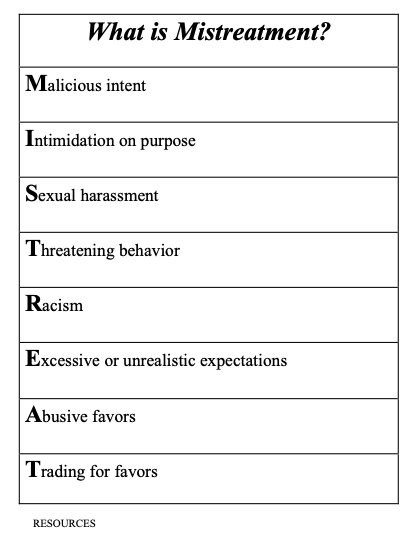

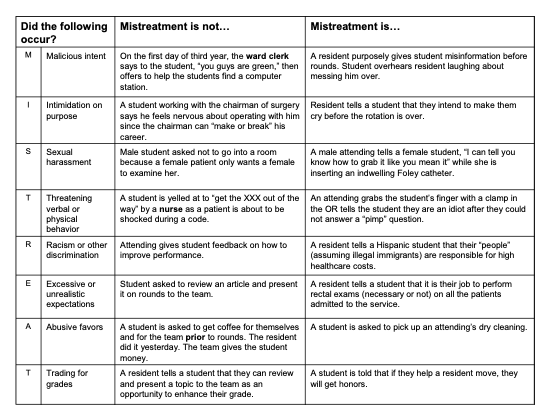
*

Image by Reddy et al, retrieved from <https://doi.org/10.15766/mep_2374-8265.9569> on 01/20/2020. Creative Commons License associated: <https://creativecommons.org/licenses/by-nc-sa/4.0/legalcode>

**Learning Environment Session Group Facilitation Script Template**

*(Introduce yourself) and I will be acting as scribe for this Clerkship Learning Environment session. As a reminder, the purpose of the Learning Environment Sessions is to elicit feedback that is both honest and actionable. These sessions came about after school data revealed our school had a greater amount of student mistreatment than the national averages. As you know, our school defines mistreatment as any behavior that is harmful or offensive to an individual student and interferes with the student’s learning. The school takes this very seriously and so, in addition to the end of clerkship evaluations and the ATM, the school, clerkship directors and student representatives have set up this student-led clerkship discussion group to attempt to identify any issues of concern so that they can be addressed in real time.*

*We want to start by establishing some ground rules for this session. First, this session is entirely anonymous and what is said here, stays here. We will not write any names of students down or attribute anything that is said during this session to a particular person. We understand you may have concerns about being easily identifiable despite not being named, particularly if you work with a preceptor or at a site that receives fewer students, but we want to reassure you that Clerkship Directors will not take any action with a given preceptor before grades are released, unless the situation merits immediate intervention. If you would rather an issue be addressed in a later block, let us know and we can document this preference in the minutes. Only in the case that someone’s physical or mental health is in imminent danger will we opt to disclose a student’s identity to an appropriate resource.*

*Second, we will aim to make this session as transparent as possible. You will be able to see what I write to make sure you are comfortable with it. I will write down everything that is said during the session and this document will be sent to both the CD and the MEO after this meeting. The CD and MEO will be accountable for following up on the concerns we bring forward. If serious issues of mistreatment come up, the MEO will send a list of themes to the ATM chair, without identifying any particular incident.*

*Third, we have found that discussing issues in the group setting can be helpful for students, as students often have shared experiences, both positive and negative, during their third year. By talking with the group, we may be able to more quickly identify problematic patterns or treatment. However, if you are more comfortable talking one-on-one about issues, you can approach us after the session, as we will stay in the room for an additional __ minutes to be available to talk. We can also be reached by (contact info available at the top of scribe document). If you have experienced mistreatment during this rotation and would like to file an ATM report or need help finding resources for support, we can help guide you. You can file an ATM report with your name or anonymously, based on your preference. If you would like for a report to be filed on your behalf, we are available for that as well.*

*Lastly, because this is meant to fix issues in the learning environment, we need to provide feedback that is truthful, actionable and relevant to your experience. Describing specific behaviors and giving concrete examples is important so that the CD and/or MEO can both identify the specific issues and fix them. Because this is anonymous, the CDs and MEO will have a limited ability to follow up with us to ask specific questions, so the more specific and actionable we can be initially, the better. As facilitators, we may ask you for more information or to brainstorm an action plan so we can get the most usable data to the CD’s and MEO. If you are not comfortable answering our follow up questions, that is fine, just please let us know. We may also not be addressing topics that you think are important – please help expand our conversation.*

**Facilitator Instructions:**

Please discuss the following topics with the group. You don’t necessarily need to go in the order listed below, but you should make sure that all or as many of these topics are covered as possible. Please follow up on any topics or student comments in which you feel you need more information to lead the group to actionable specific feedback to deliver to the clerkships. For facilitation tips, please see the

*Script:*

*Now I will ask you about several different components of the learning environment. After I ask about a topic, please share any thoughts, comments, or issues that you feel have impacted your experience. We’ll make sure that everyone who wants to share has a chance to contribute in each area before we move on to the next topic. As we scribe, please feel free to suggest edits to what we are writing so we can capture your feedback in a way that is both accurate and comfortable for you.*

• Unfair treatment: *If an issue arises with personnel please quantify the number of students who felt this way and get concrete details if possible (as the student is comfortable)*

- *Did anyone treat you in a way you felt was harmful or offensive and interfered with your learning?*
- *Did you see anyone treat another student in a way you felt was harmful or offensive and interfered with your learning?*
- *Were you put into any situations where you felt you were publicly humiliated or embarrassed?*
- *Were you put into any situations where you felt discriminated or treated unfairly due to biases regarding race, gender, sexuality?*
- *Were you put into any situations where you felt discriminated against or treated unfairly due to the hierarchical nature of medical training?*
- *Did you feel you were put in a situation where you didn’t have adequate supervision?*
- *Did you see anyone treat a staff member, resident, faculty or patient in a way you felt was harmful or offensive and interfered with your learning.*

• Capture the preceptors that students aren’t sending clinical student evaluation forms (CSEFs) to or are avoiding

- *Are there any preceptors to whom you did not submit a CSEF due to fear of unfair grading?*
  - *Was this due to a personal experience or word-of-mouth from other students?*
- *Are there any preceptors who you felt used (or feared they would use) grading and/or other forms of assessment in a punitive, harassing, or discriminatory manner?*

• Any course communication issues/staff issues

- *Did any of the administrative staff, clerkship coordinators or directors communicate with you in a way you felt was harmful, offensive or discriminatory?*

• Didactic faculty issues

- *Did any of the faculty communicate or treat you in a way you felt was harmful, offensive or discriminatory?*

• Site specific issues

- *Did anyone treat you in a way you felt was harmful or offensive and interfered with your learning at any of your sites outside of BMC?*

• Curricular learning environment

- *Are there other issues with the learning environment (“curricular events”) which may not meet the definition of mistreatment but may raise concerns for a student. Curricular events refer to concerns that occur during medical student specific didactics or teaching events outside of the clinical environment*

• Anyone who was really great

- *Did anyone go above and beyond to help teach you or the group? To help you get oriented? Any best practices?*
- *Are there any residents you feel should be recognized as exceptional by their residency directors?*

**Learning Environment Session Facilitator Documentation Template**

**Clerkship: *****

**Block: *****

**Unfair treatment**

**Preceptors students aren’t sending clinical student evaluation forms (CSEFs) to or are avoiding**

**Any course communication issues/staff issues**

**Didactic faculty issues**

**Site specific issues**

**Curricular learning environment**

**Anyone who was really great**

**Other**

**After Session Instructions**

Please scribe minutes *fully* to be sent to the Medical Education Office (MEO) Deans. They will review the complete document and will file the forms to be reviewed annually with the clerkship directors.

Then, edit the document to note non-mistreatment topics that have been discussed, but erase the contents of the conversation. Send that truncated version to the Clerkship Directors and cc in the MEO Deans.

Clerkship Directors do not change didactics or parts of the course based on LES minutes because they represent the opinions of 1 block of students. However, they can change issues with logistics immediately and are responsible to respond to issues to mistreatment in real-time. If the Clerkship Directors are interested in knowing what was said on a curricular topic, they can ask the MEO for more detailed notes and they will review the detailed notes once a year.

**Facilitation Tips**

To be an effective facilitator, it’s important to lay the groundwork for a fruitful conversation (ie the preventions) and then be able to act in the moment to steer the conversation (ie the interventions).

Preventions

- **Preparation**: Are you prepared for the session? Have you gone over the content material and questions you would like to ask? What kind of group feel would you like to cultivate?
- **Service:** it is important to consider what kind of service you would like to provide to the group. At times, such as during LES sessions, you may not have an end-goal in sight but want to provide a service to the group by opening up a space for conversation.
- **Lean into discomfort**: The conversation may veer in uncomfortable directions based on students’ experiences of mistreatment. This may create discomfort for you personally to talk about clinicians you have worked with. Burying that discomfort will not make it go away, so we urge you to lean into it and confront it mentally prior to the session.
- **Midwife:** the idea of being a midwife to the conversation is that you are not delivering and pushing the conversation, but rather catching it based on what is said. A midwife helps by assessing their patients where they are and tries to push them to their ultimate goal. Remember- it is important to distinguish who is doing the work.
- **Clear outcomes**: While the conversation may take its own twists and turns, it is important to maintain clear outcomes. One way to do this effectively is to push the group to think of their own solutions by prompting them such as “I’m sorry you experienced this, what would you have wanted to see happen at that time?”
- **Agenda:** Move through the agenda to keep the session going. It is OK for the group to not having very much to contribute at different times.
- **Ground rules for psychological safety**: It is important to create a culture of safety where it is better to speak up about something one is unsure of rather than to stay quiet about a potentially unsafe practice. Some ground rules to consider- what is said in here stays in here, use I statements, active listening, support one another, no judgment, etc.
- **Roles**: Remember your roles, which may be different at times as you transition from student to facilitator. Sometimes, it is helpful to determine whether mistreatment has happened by remember our clinical responsibilities and expectations as set out by our curriculum.
- **Agreement to group rules**: Rather than asking for agreement, check for disagreement. Do this by asking, “Is there anything anyone wants to change about our ground rules?”

Interventions

- **Boomerang**: To prevent a conversation from getting heated, you can have it come back to you rather than developing between two people
- **Ask/say:** “What’s going on”: this is a non-threatening way to act curious about a potentially decompensating conversation
- **Enforce process agreements**: Another technique includes referencing group agreement rules to try to get the group centered and focused on its goal
- **Regain focus**: It is important to note when the conversation is deviating from its goal (e.g. to when students are focusing more on the assessment form than on mistreatment). You can call it out or move on to the next prompting question to try to head back to your structure/regain focus.
- **Accept/Legitimize**: When a student has brought up an uncomfortable experience and is met with resistance sometimes it is best to air on the side of legitimizing and validating that experience. Then follow it up by pushing the group to come up with their own solutions.

**References**

AAMC. “2019 All Schools Summary Report.” AAMC. Accessed Nov 2019. Available at: <https://www.aamc.org/system/files/2019-08/2019-gq-all-schools-summary-report.pdf>

Agency for Healthcare Research and Quality. Creating Psychological Safety in Teams. Accessed Dec 2019. Available at: <https://www.ahrq.gov/sites/default/files/wysiwyg/evidencenow/tools-and-materials/psychological-safety.pdf>

Chung MP, Thang CK, Vermillion M, Fried JM, Uijtdehaage S. Exploring medical students' barriers to reporting mistreatment during clerkships: a qualitative study. *Med Educ Online*. 2018;23(1):1478170. DOI: [10.1080/10872981.2018.1478170](https://doi.org/10.1080/10872981.2018.1478170)

Feldman NL, Lewis JL, Patel CK, et al. The other side of medical student mistreatment: teaching cultural competency across the generational divide. *MedEdPORTAL*. 2019;15:10847. [https://doi.org/10.15766/mep_2374-8265.10847](about:blank)

Reddy S, Ogden P, Arora V, et al. Is it mistreatment? Mistreatment education for medical students entering clinical training. *MedEdPORTAL*. 2013;9:9569. <https://doi.org/10.15766/mep_2374-8265.9569>
