# Supplementary material for: Training Medical Students as Peer-Facilitators to Identify Medical Student Mistreatment in the Clerkship Year
Source: MedEdPORTAL. 2021 Sep 27;17:11185. doi: 10.15766/mep_2374-8265.11185 (PMC8473588; doi:10.15766/mep_2374-8265.11185)
Supplement: Supplementary file 1 — Facilitator Application.docxFacilitator Orientation.pptxMidyear Facilitator Training.pptxFacilitator Packet for Midyear Training.docxFacilitator Training Role-Play Activity.docxMidyear Training Evaluation.docx [file mep_2374-8265.11185-s001.zip › E. Facilitator Training Role-Play Activity.docx]

**Facilitator Training Role Play Activity**

Form groups of 4 people. There are 4 scenarios you will role play. Start off by having one person be the facilitator, one be student 1, one be student 2 and one be student 3 in the first case. In the second case everyone should move to the next role (so the facilitator will now be student 1, student 1 will now be student 2, etc.). Rotate like this through all the cases so that everyone has an opportunity to be a facilitator. When you are the facilitator, you should not look at the scenario student scripts!

Facilitators should start by introducing the session/setting ground rules and then should go through the script and ask questions like you would if you were facilitating an actual session.

Students should play the roles they are assigned. The descriptions are brief so you can also draw from your own experiences to help.

**Intros**: ~1 min → “Stop Action” to give feedback on intro
**Role play:** ~5 mins
**Feedback**: ~4 mins (start with facilitator’s reflection)

**Scenario 1: Surgery clerkship**

Student 1 Script: When asking about unfair treatment, you say “I’m not sure that this is actually harmful treatment, but I am constantly encountering dirty jokes when working with the residents. Honestly, they just think they are joking around but I kind of think they are offensive.” If asked for more specifics you can say, “You’re not going to write me up for that, are you? I definitely don’t want it coming back to me or affecting my grade- I’m thinking about going into general surgery.”

Student 2 Script: You are reluctant to talk at first, and you should look very disinterested. Eventually you will share that there have been some issues about work hours but you’re not sure if you should log that or not, and that you feel really burned out.

Student 3 Script: You are at an away site with medical students from other institutions rotation there as well. You’ve been having a hard time with the other med students. They keep saying things like, “Well you’re from BU, so you must know everything”

**Scenario** **2: Ob/Gyn clerkship**

.

Student 1 Script: You are a male student who has not been included in a lot of appointments because of your gender. You tried to advocate for yourself and was told sternly, *“A lot of patients aren’t comfortable with males and I have to put their needs first.”*

Student 2 Script: Feels like the Clinical Student Evaluation Forms are not an accurate representation of how they are doing. Has sent 20+ forms and only gotten back 2, of which one graded them down for professionalism.

Student 3 Script: Bleary-eyed. Just had some overnights this past weekend and is now readjusting to days so very exhausted and very worried about the upcoming shelf exam.

**Scenario 3: Medicine clerkship**

Student 1 Script: You are always on very large teams so it’s hard to contribute to patient care meaningfully. You feel like every time you get close to getting involved with the team, you have to go to small group didactics. On rounds, you are only given feedback about patient interaction and everyone praises your ability to connect with patients. You’ve noticed, though, that your male colleagues are pressed more on their management plans and are given the opportunity to practice putting in orders and taking consults on their own. You’ve tried to bring this up, but you were just told that timing is not on your side.

Student 2 Script: You are really worried about caliber of medicine at outside hospital, and are unsure how to bring this up and who to bring this up to

Student 3 Script: You are very tired from the 6 days/week schedule. You have missed out on family gatherings and doctors’ appointments and are feeling very burned out.

**Scenario 4: Psychiatry clerkship**

Student 1 Script: You were told to take a consult on your own with a patient known to be physically aggressive. You did not feel like you had any choice in the matter and did not know how to prepare for that experience and keep yourself safe. Nothing happened, but it could have.

Student 2 Script: You are having a great experience at an outside hospital but had one encounter where you were interviewing a patient and the patient kept asking about personal details of your life. For example, they saw your wedding ring and wanted to know more about whether you were married, etc. You felt uncomfortable and you were unsure how to redirect the conversation because you didn’t want to affect the trust of the patient.

Student 3 Script: You are really enjoying all of the ‘reading time’ you get. You feel much better than last block when they were on Medicine, but you are not sure how much learning you are really getting.
