# Supplementary material for: Training Medical Students as Peer-Facilitators to Identify Medical Student Mistreatment in the Clerkship Year
Source: MedEdPORTAL. 2021 Sep 27;17:11185. doi: 10.15766/mep_2374-8265.11185 (PMC8473588; doi:10.15766/mep_2374-8265.11185)
Supplement: Supplementary file 1 — Facilitator Application.docxFacilitator Orientation.pptxMidyear Facilitator Training.pptxFacilitator Packet for Midyear Training.docxFacilitator Training Role-Play Activity.docxMidyear Training Evaluation.docx [file mep_2374-8265.11185-s001.zip › F. Midyear Training Evaluation.docx]

**Learning Environment Sessions Training Evaluation**

**Demographic questions**

Age range

Gender

**Please circle a number on the 1-5 scale for each of the following statements.**

1. I understand the definition of mistreatment and the most common types, as well as sources of mistreatment in medical education.

0 1 2 3 4

              Do Not Agree       Neutral     Highly Agree

1. I believe that medical education should be free of learner mistreatment.

0 1 2 3 4

              Do Not Agree       Neutral     Highly Agree

1. I believe that peer-led sessions offer an important avenue to help students process experiences of mistreatment.

0 1 2 3 4

              Do Not Agree       Neutral     Highly Agree

1. I feel confident identifying different types of mistreatment, as well as their possible sources.

0 1 2 3 4

              Do Not Agree       Neutral     Highly Agree

1. I understand different barriers that students experience to reporting mistreatment.

0 1 2 3 4

              Do Not Agree       Neutral     Highly Agree

1. I feel confident facilitating feedback sessions with my peers that create a space for psychological safety.

0 1 2 3 4

              Do Not Agree       Neutral     Highly Agree

1. I have strategies to support my peers who have experienced mistreatment.

0 1 2 3 4

              Do Not Agree       Neutral     Highly Agree

1. I feel confident navigating the mechanisms for reporting mistreatment at Boston University School of Medicine

0 1 2 3 4

              Do Not Agree       Neutral     Highly Agree

1. I trust the reporting mechanisms for mistreatment at Boston University School of Medicine.

0 1 2 3 4

              Do Not Agree       Neutral     Highly Agree

1. I feel confident about documenting feedback sessions to best represent my peers’ views and experiences.

0 1 2 3 4

              Do Not Agree       Neutral     Highly Agree

**My top 2 takeaways from this workshop were:**

**Suggestions to improve this training:**

**Suggestions to improve Learning Environment Sessions:**
