# Supplementary material for: Training Medical Students as Peer-Facilitators to Identify Medical Student Mistreatment in the Clerkship Year
Source: MedEdPORTAL. 2021 Sep 27;17:11185. doi: 10.15766/mep_2374-8265.11185 (PMC8473588; doi:10.15766/mep_2374-8265.11185)
Supplement: Supplementary file 1 — Facilitator Application.docxFacilitator Orientation.pptxMidyear Facilitator Training.pptxFacilitator Packet for Midyear Training.docxFacilitator Training Role-Play Activity.docxMidyear Training Evaluation.docx [file mep_2374-8265.11185-s001.zip › A. Facilitator Application.docx]

Learning Environment Session Facilitators

Student volunteer availability

1. Email address
2. Name
3. Please select which clerkships you have completed. (Include those you are currently taking as completed) *Check all that apply.*

|  | Completed | Not Completed | Not Taking |
| --- | --- | --- | --- |
| Medicine |  |  |  |
| Surgery |  |  |  |
| Ob/Gyn |  |  |  |
| Psychiatry |  |  |  |
| Pediatrics |  |  |  |
| Family Medicine |  |  |  |
| Neurology |  |  |  |
| Emergency Medicine |  |  |  |
| Radiology |  |  |  |


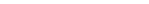

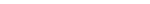


1. In 2-4 sentences, why are you motivated to participate as a leader of the Learning Environment sessions?
2. Field into which you will most likely apply for residency?
