# Supplementary material for: Training Medical Students as Peer-Facilitators to Identify Medical Student Mistreatment in the Clerkship Year
Source: MedEdPORTAL. 2021 Sep 27;17:11185. doi: 10.15766/mep_2374-8265.11185 (PMC8473588; doi:10.15766/mep_2374-8265.11185)
Supplement: Supplementary file 1 — Facilitator Application.docxFacilitator Orientation.pptxMidyear Facilitator Training.pptxFacilitator Packet for Midyear Training.docxFacilitator Training Role-Play Activity.docxMidyear Training Evaluation.docx [file mep_2374-8265.11185-s001.zip › B. Facilitator Orientation.pptx]

## Slide 1
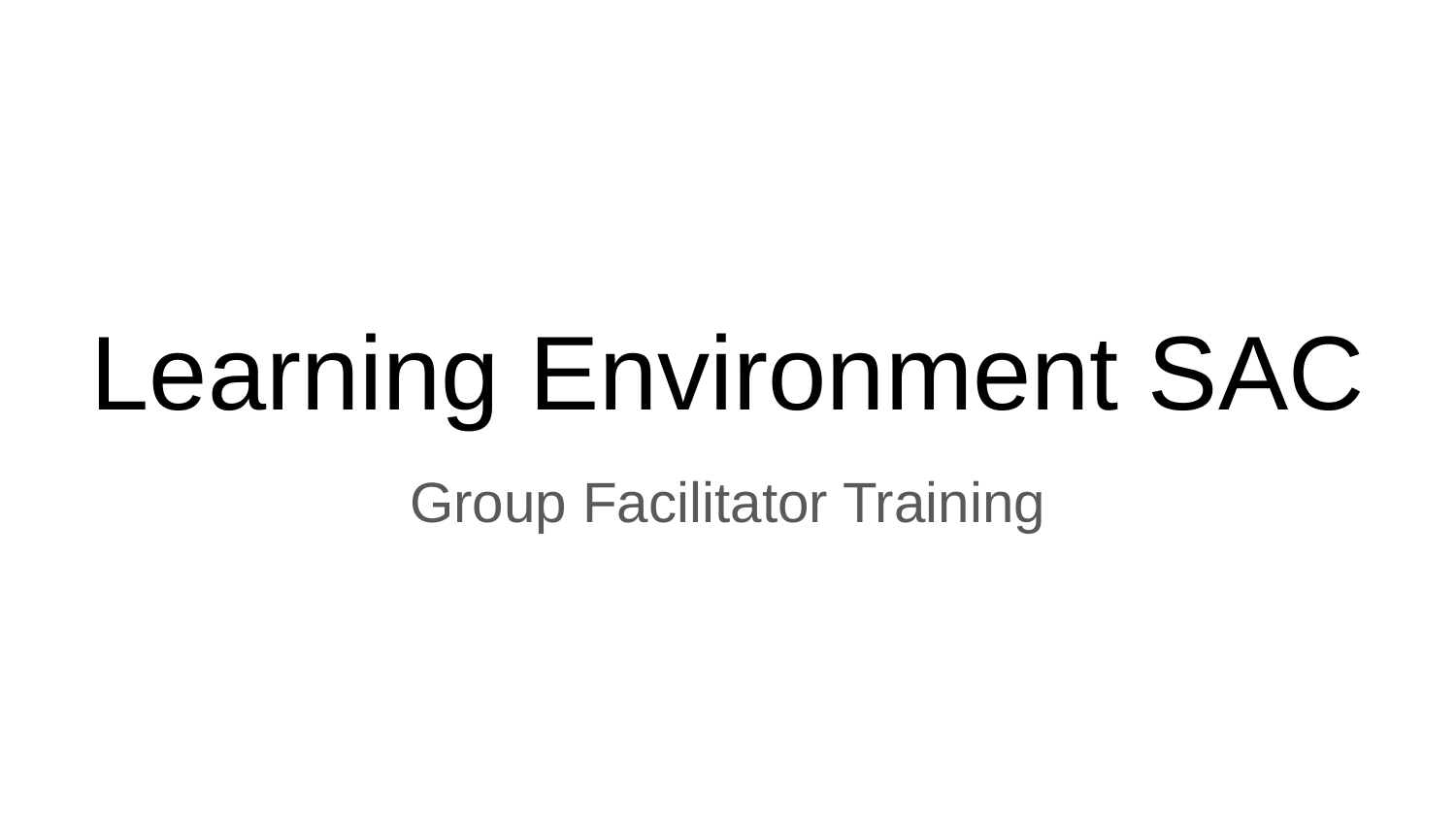

# Learning Environment SAC
Group Facilitator Training

## Slide 2
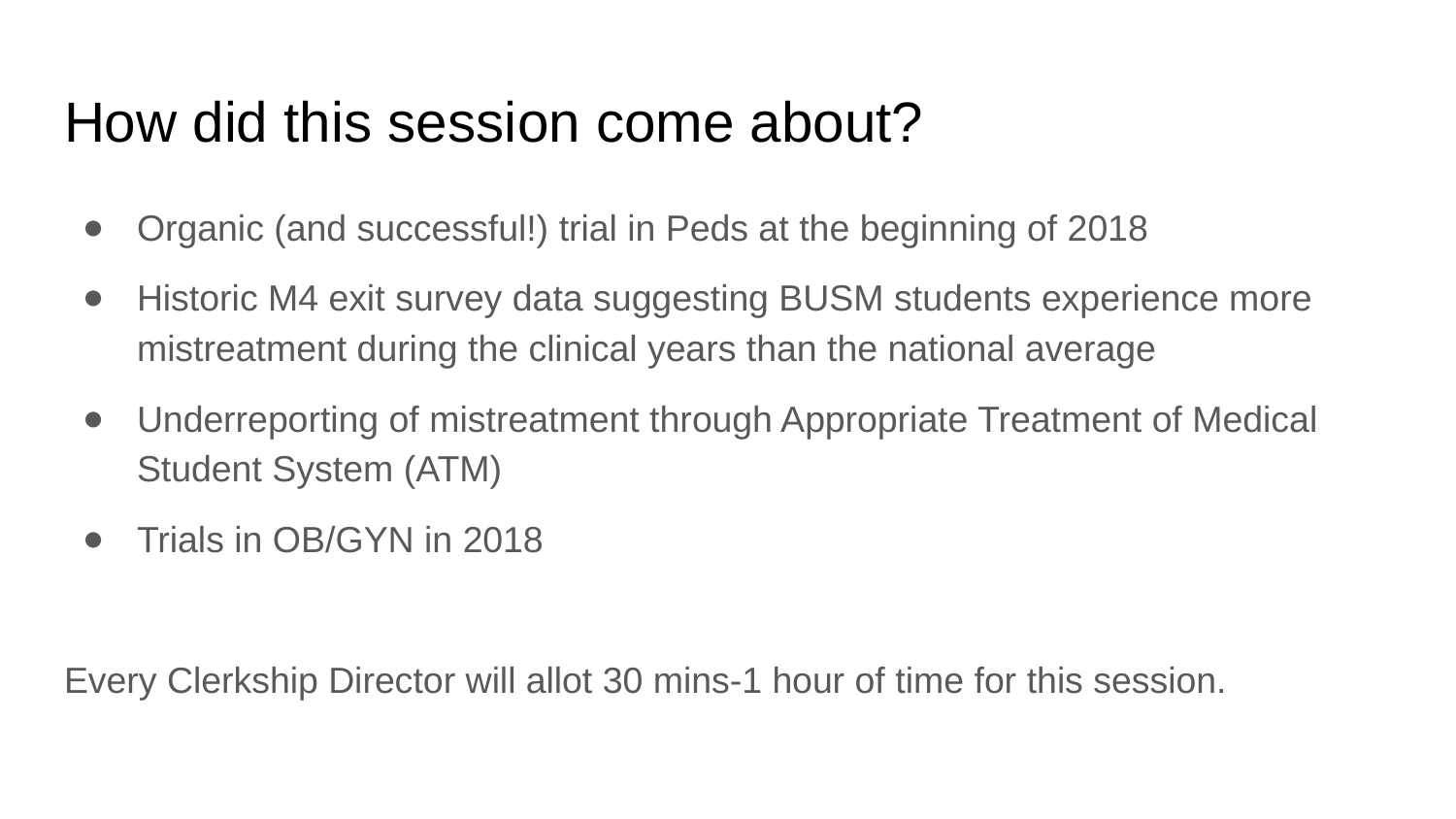

# How did this session come about?
Organic (and successful!) trial in Peds at the beginning of 2018
Historic M4 exit survey data suggesting BUSM students experience more mistreatment during the clinical years than the national average
Underreporting of mistreatment through Appropriate Treatment of Medical Student System (ATM)
Trials in OB/GYN in 2018
Every Clerkship Director will allot 30 mins-1 hour of time for this session.

## Slide 3
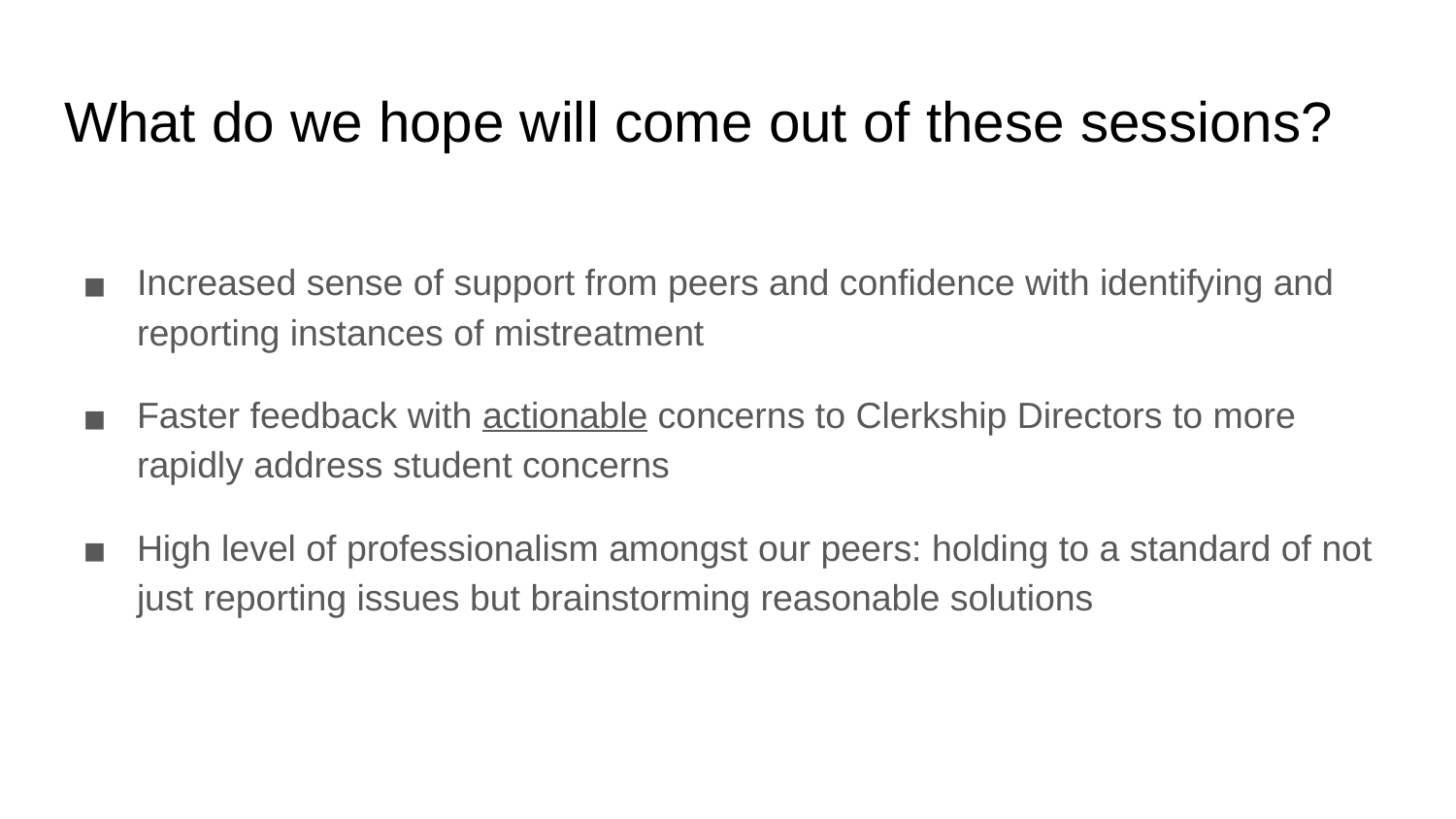

# What do we hope will come out of these sessions?
Increased sense of support from peers and confidence with identifying and reporting instances of mistreatment
Faster feedback with actionable concerns to Clerkship Directors to more rapidly address student concerns
High level of professionalism amongst our peers: holding to a standard of not just reporting issues but brainstorming reasonable solutions

## Slide 4
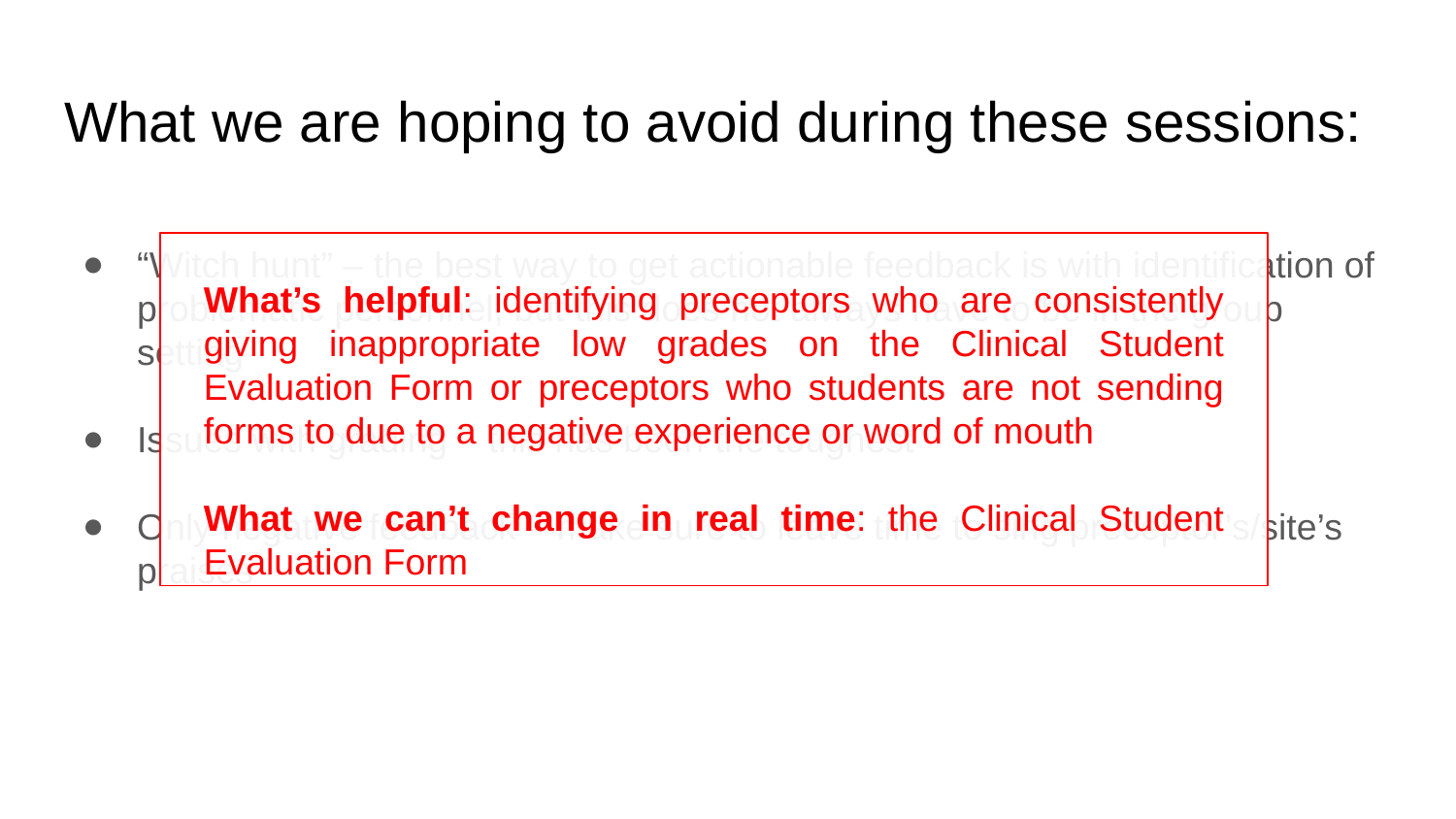

# What we are hoping to avoid during these sessions:
“Witch hunt” – the best way to get actionable feedback is with identification of problematic personnel, but this does not always have to be in the group setting
Issues with grading – this has been the toughest
Only negative feedback – make sure to leave time to sing preceptor’s/site’s praises
What’s helpful: identifying preceptors who are consistently giving inappropriate low grades on the Clinical Student Evaluation Form or preceptors who students are not sending forms to due to a negative experience or word of mouth
What we can’t change in real time: the Clinical Student Evaluation Form

## Slide 5
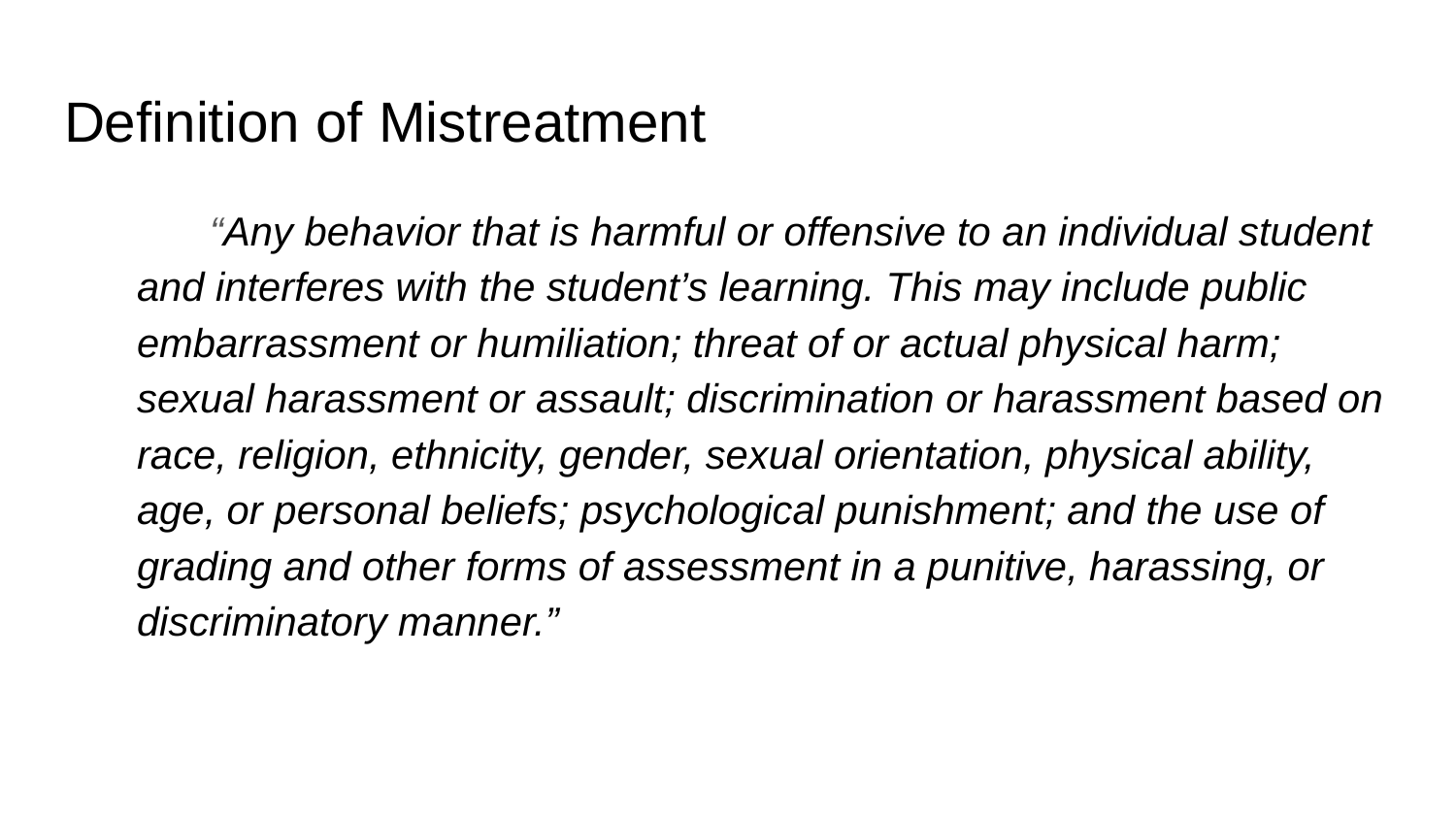

# Definition of Mistreatment
“Any behavior that is harmful or offensive to an individual student and interferes with the student’s learning. This may include public embarrassment or humiliation; threat of or actual physical harm; sexual harassment or assault; discrimination or harassment based on race, religion, ethnicity, gender, sexual orientation, physical ability, age, or personal beliefs; psychological punishment; and the use of grading and other forms of assessment in a punitive, harassing, or discriminatory manner.”

## Slide 6
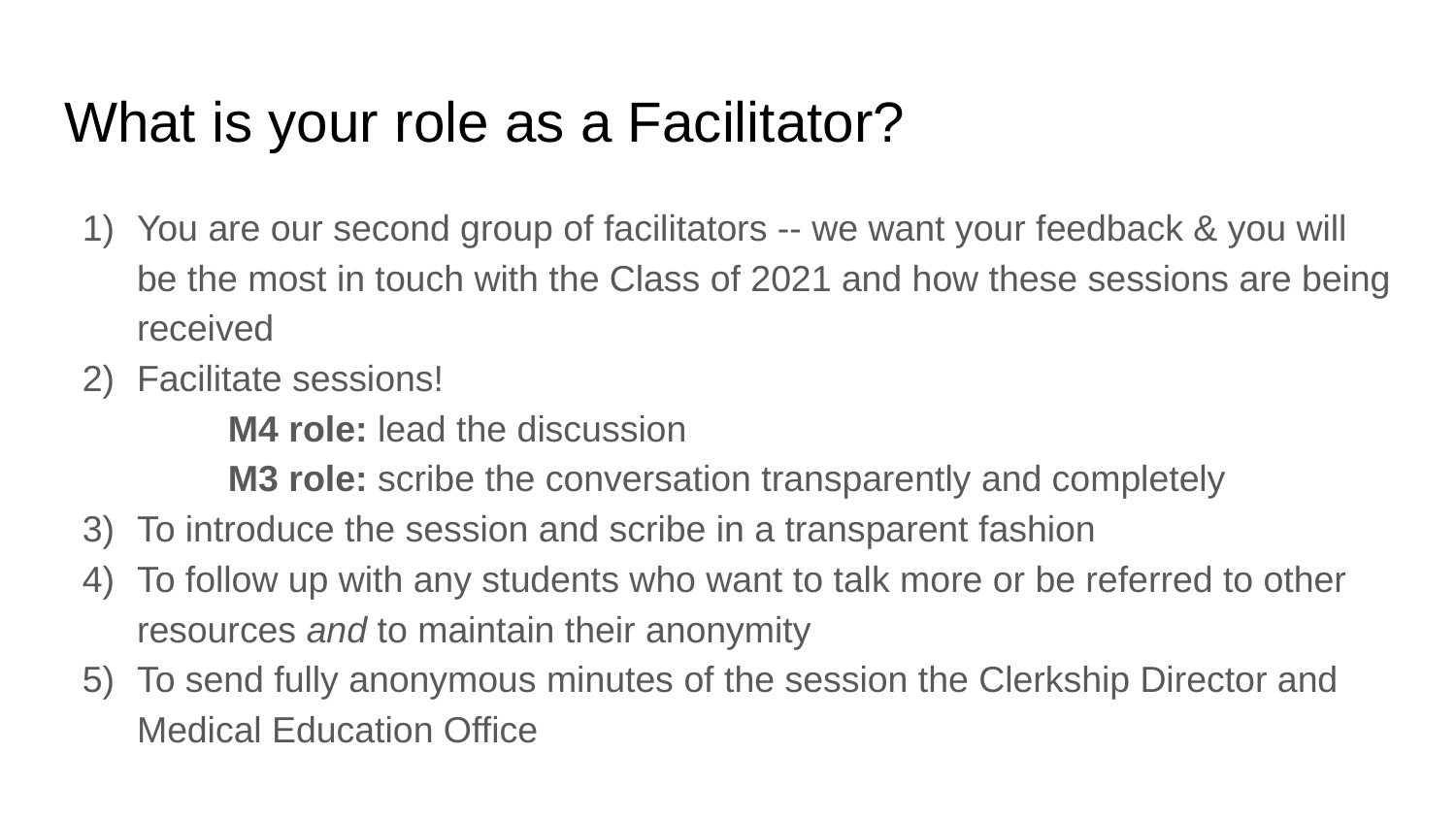

# What is your role as a Facilitator?
You are our second group of facilitators -- we want your feedback & you will be the most in touch with the Class of 2021 and how these sessions are being received
Facilitate sessions!
	M4 role: lead the discussion
	M3 role: scribe the conversation transparently and completely
To introduce the session and scribe in a transparent fashion
To follow up with any students who want to talk more or be referred to other resources and to maintain their anonymity
To send fully anonymous minutes of the session the Clerkship Director and Medical Education Office
Your contact information will be listed for students at the start of each session.

## Slide 7
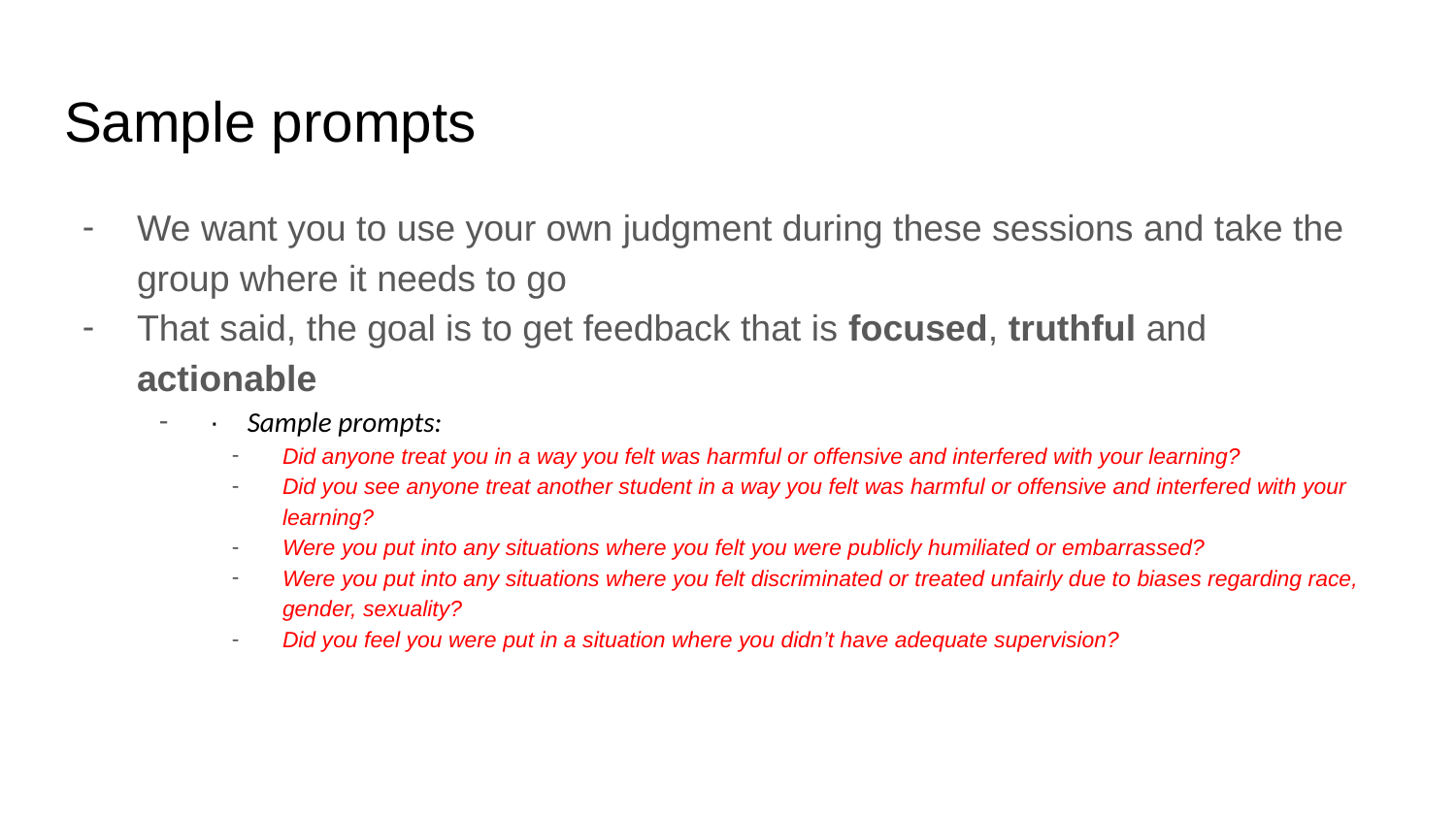

# Sample prompts
We want you to use your own judgment during these sessions and take the group where it needs to go
That said, the goal is to get feedback that is focused, truthful and actionable
· Sample prompts:
Did anyone treat you in a way you felt was harmful or offensive and interfered with your learning?
Did you see anyone treat another student in a way you felt was harmful or offensive and interfered with your learning?
Were you put into any situations where you felt you were publicly humiliated or embarrassed?
Were you put into any situations where you felt discriminated or treated unfairly due to biases regarding race, gender, sexuality?
Did you feel you were put in a situation where you didn’t have adequate supervision?

## Slide 8
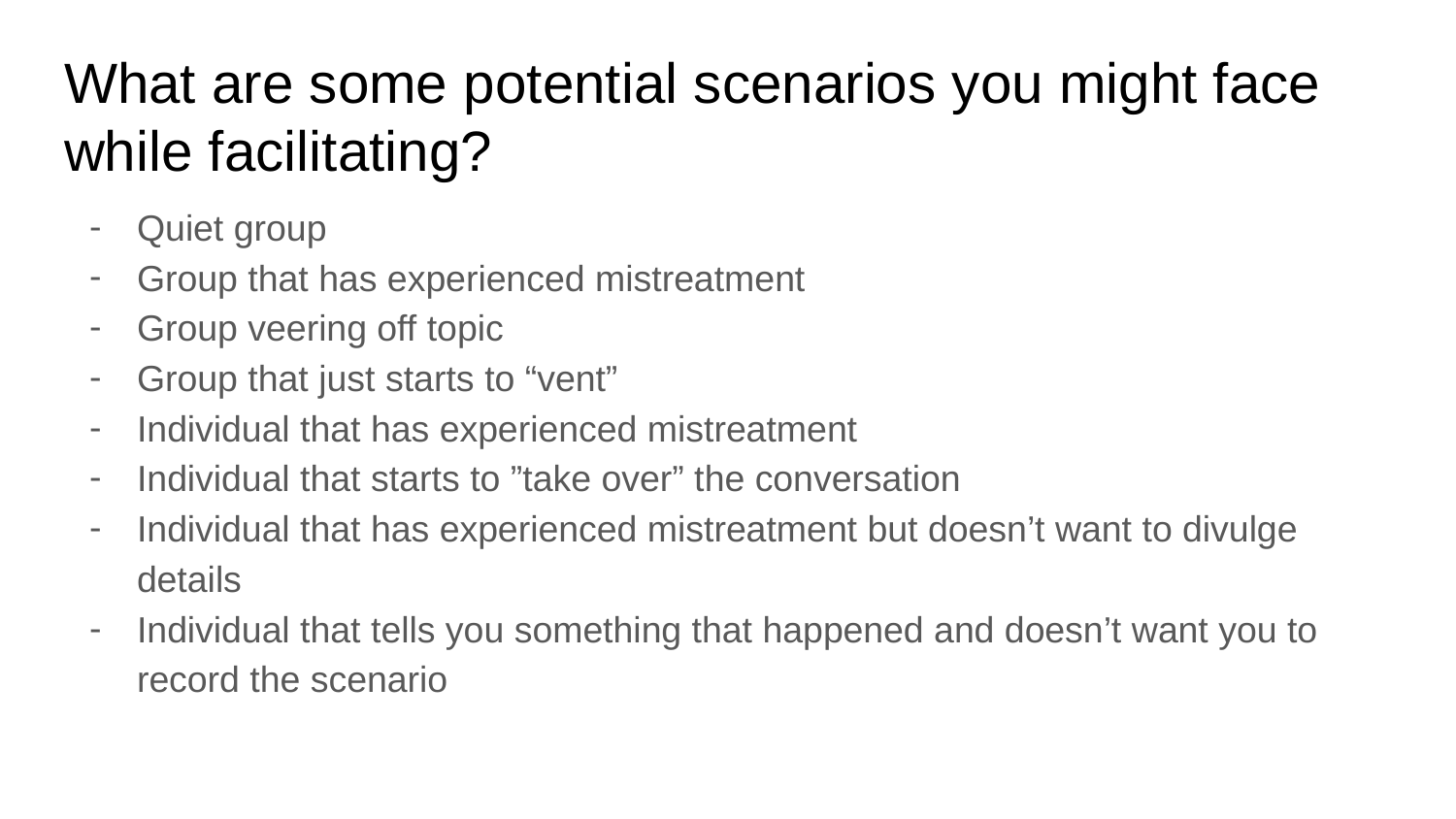

# What are some potential scenarios you might face while facilitating?
Quiet group
Group that has experienced mistreatment
Group veering off topic
Group that just starts to “vent”
Individual that has experienced mistreatment
Individual that starts to ”take over” the conversation
Individual that has experienced mistreatment but doesn’t want to divulge details
Individual that tells you something that happened and doesn’t want you to record the scenario

## Slide 9
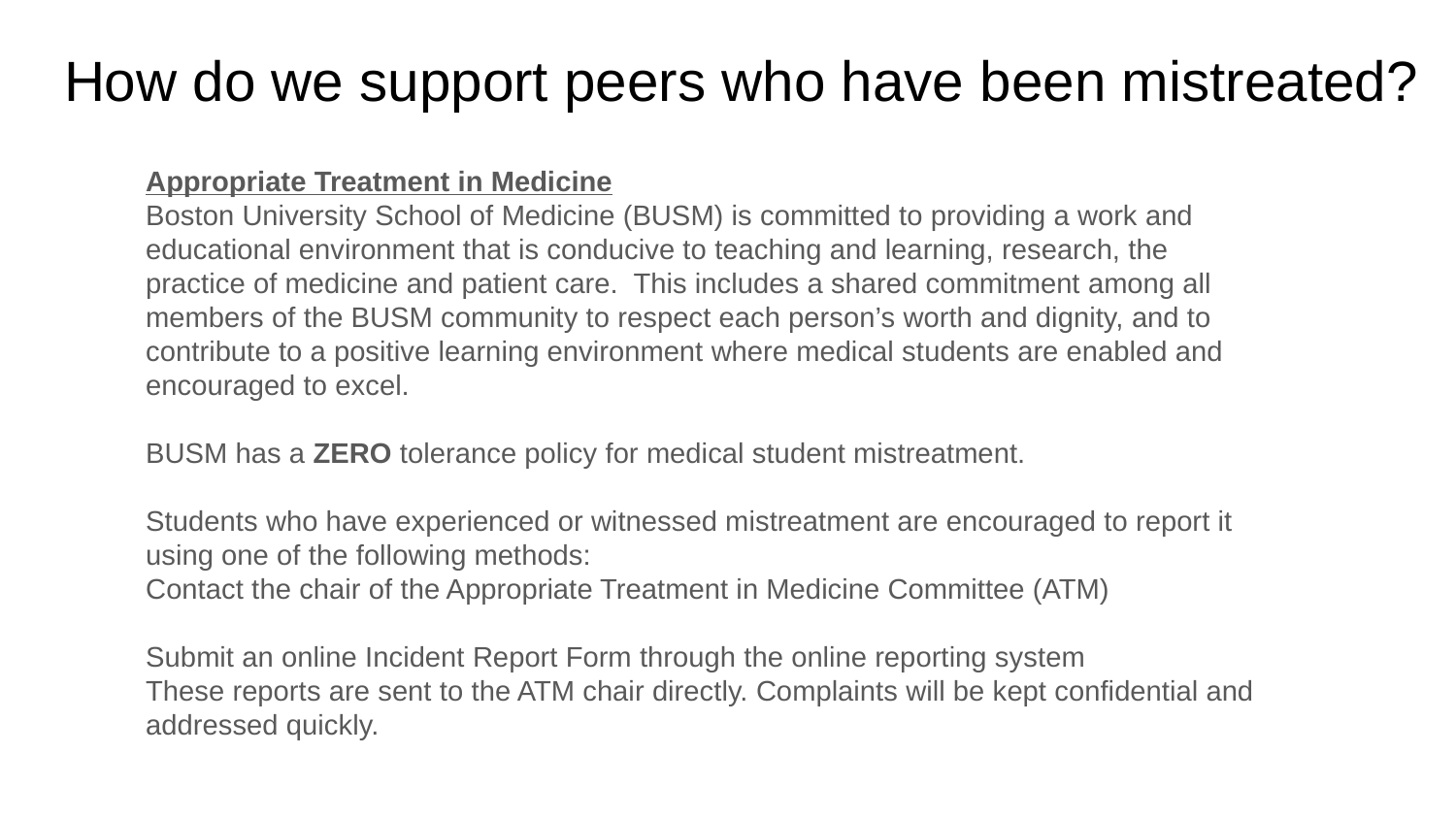

# How do we support peers who have been mistreated?
Appropriate Treatment in Medicine
Boston University School of Medicine (BUSM) is committed to providing a work and educational environment that is conducive to teaching and learning, research, the practice of medicine and patient care. This includes a shared commitment among all members of the BUSM community to respect each person’s worth and dignity, and to contribute to a positive learning environment where medical students are enabled and encouraged to excel.
BUSM has a ZERO tolerance policy for medical student mistreatment.
Students who have experienced or witnessed mistreatment are encouraged to report it using one of the following methods:
Contact the chair of the Appropriate Treatment in Medicine Committee (ATM)
Submit an online Incident Report Form through the online reporting system
These reports are sent to the ATM chair directly. Complaints will be kept confidential and addressed quickly.

## Slide 10
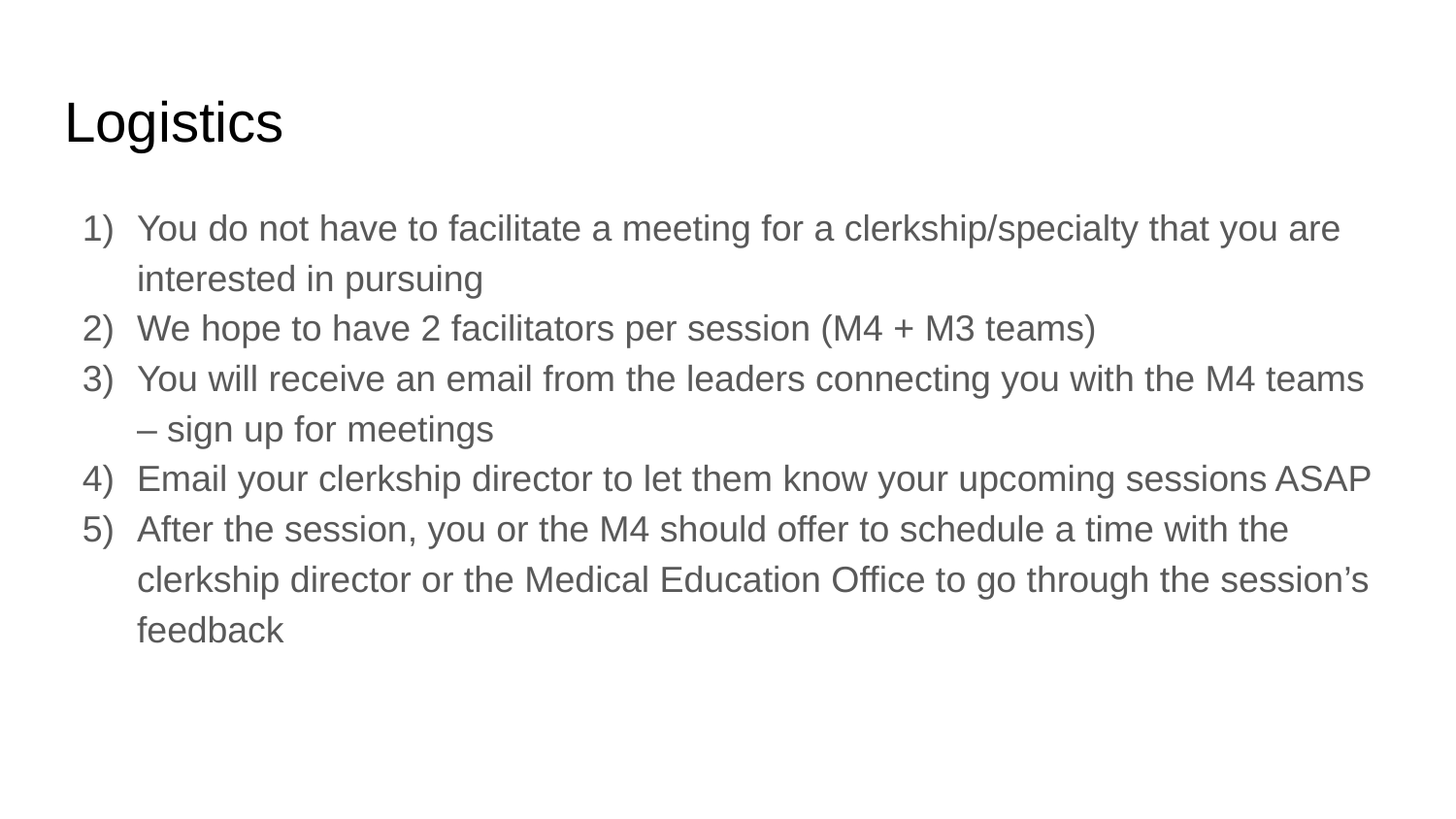

# Logistics
You do not have to facilitate a meeting for a clerkship/specialty that you are interested in pursuing
We hope to have 2 facilitators per session (M4 + M3 teams)
You will receive an email from the leaders connecting you with the M4 teams – sign up for meetings
Email your clerkship director to let them know your upcoming sessions ASAP
After the session, you or the M4 should offer to schedule a time with the clerkship director or the Medical Education Office to go through the session’s feedback

## Slide 11
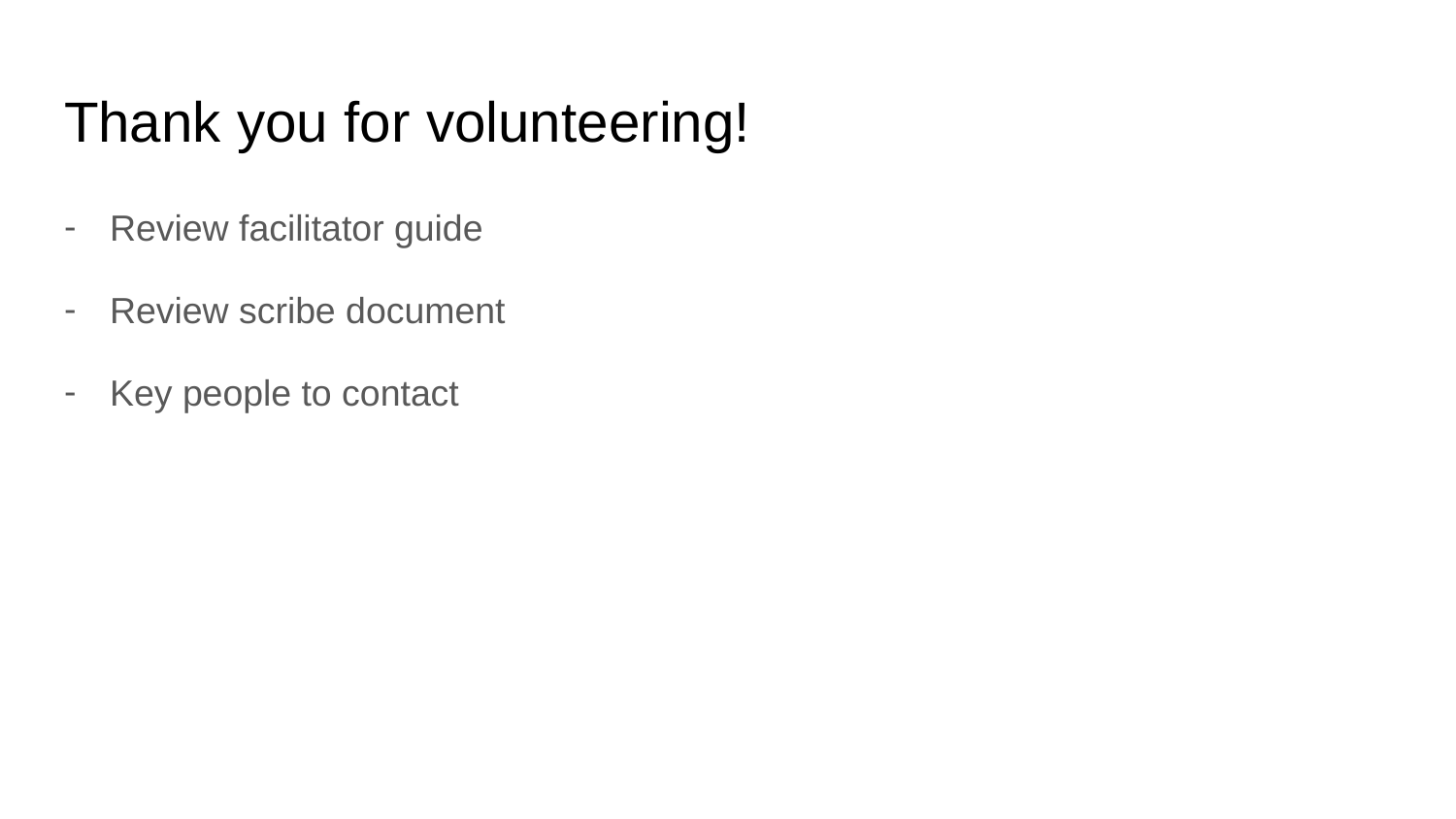

# Thank you for volunteering!
Review facilitator guide
Review scribe document
Key people to contact
